# Cytotoxicity, acute and sub-chronic toxicities of the leaves of *Bauhinia thonningii* (Schumach.) Milne-Redh. (Caesalpiniaceae)

**DOI:** 10.1186/s12906-023-04172-9

**Published:** 2023-09-27

**Authors:** Valaire Y. Matieta, Armelle T. Mbaveng, Guy R. Sado Nouemsi, Simplice B. Tankeo, Gabriel T. Kamsu, Paul Nayim, Alain M. Lannang, İlhami Çelik, Thomas Efferth, Victor Kuete

**Affiliations:** 1 Department of Biochemistry, Faculty of Science, University of Dschang, Dschang, Cameroon; 2 Department of Pharmaceutical Biology, Institute of Pharmaceutical and Biomedical Sciences, University of Mainz, Staudinger Weg 5, 55128 Mainz, Germany; 3 Department of Chemistry, Faculty of Science, University of Maroua, Maroua, Cameroon; 4 Department of Chemistry, Faculty of Science, Eskişehir Technical University, Eskisehir, Turkey

**Keywords:** 6,8-C-dimethylkaempferol-3-methyl ether, Cancer, Cytotoxicity, Phytochemistry, *Bauhinia thonningii*, Toxicity

## Abstract

**Background:**

*Bauhinia thonningii* is a plant traditionally used against many human diseases such as gastric ulcers, fever, inflammations, coughs, dysentery, diarrhea, and malaria. In the present investigation, the cytotoxicity of methanol extract of *Bauhinia thonningii* leaves (BTL), fractions and the isolated phytoconstituents was determined in a panel of 9 human cancer cell lines including drug sensitive and multidrug-resistant (MDR) phenotypes. The acute and sub-chronic oral toxicity of BTL was investigated as well.

**Methods:**

Compounds were isolated using chromatographic techniques while their chemical structures were determined using spectroscopic methods. The resazurin reduction assay (RRA) was used to evaluate the cytotoxicity of samples, propidium iodide (PI) for apoptosis, 5,5′,6,6′-tetrachloro-1,1′,3,3′-tetraethylbenzimidazolylcarbocyanine iodide (JC-1) staining for mitochondrial membrane potential (MMP) analysis, 2´,7´-dichlorodihydrofluoresceine diacetate (H2DCFH-DA) staining for the quantification of reactive oxygen species (ROS), whereas Caspase Glo assays were combined by means of flow cytometry. Furthermore, the toxicological investigations were performed as recommended by the Organization for Economic Cooperation and Development (OECD).

**Results:**

The botanicals as well as 6-C-methylquercetin-3,7-dimethyl ether (2), quercetin-3-*O*-_L_-rhamnopyranoside (5), quercetin-3-*O-β*-glucopyranoside (6), 6,8-C-dimethylkaempferol 3,7-dimethyl ether (7), and 6,8-C-dimethylkaempferol-3-methyl ether (8) had promising cytotoxic effects in the 9 tested cancer cell lines. The IC_50_ values below 20 µg/mL (botanicals) or 10 µM (compounds) on at least 1/9 tested cancer cell lines were considered. The best cytotoxic effects with IC_50_ values below 5 µM were achieved with compounds 7 against CEM/ADR5000 leukemia cells (2.86 µM) and MDA-MB-231-*pcDNA* breast adenocarcinoma cells (1.93 µM) as well as 8 against CCRF-CEM leukemia cells (3.03 µM), CEM/ADR5000 cells (2.42 µM), MDA-MB-231-*pcDNA* (2.34 µM), and HCT116 *p53*^*−/−*^ cells (3.41 µM). BTL and compound 8 induced apoptotic cell death in CCRF-CEM cells through caspase activation, alteration of MMP, and increased ROS production. BTL did not cause any adverse effects in rats after a single administration at 5000 mg/kg or a repeated dose of 250 mg/kg body weight (b. w.).

**Conclusion:**

*Bauhinia thonningii* and its constituents are sources of cytotoxic drugs that deserve more in-depth studies to develop novel antiproliferative phytomedicine to fight cancer including resistant phenotypes.

**Supplementary Information:**

The online version contains supplementary material available at 10.1186/s12906-023-04172-9.

## Background

Cancer remains one of the world’s biggest threats with an alarming mortality rate placing it among the major categories of public health problems. Malignancies were responsible for around 10 million deaths worldwide in 2020 [1]. The mortality rate in Africa is estimated at up to 700,000 deaths/year [1]. According to forecasts, cancer mortality will increase considerably reaching a milestone of one million deaths per year by 2030 in case of absence of drastic and conclusive measures [2]. Its recessive resistance to current treatments such as chemotherapy, radiotherapy, and surgery is due to the ability of malignant cells to form tumors with metastatic potential and mechanisms of resistance to standard chemotherapeutics [3]. Medicinal plants are proving to be a potential source for their widespread use in the traditional medicine with over 80% of people in Sub-Saharan Africa resorting to medicinal plant treatments [2, 4]. In addition, plants have been shown in numerous studies to be a potential source of bioactive molecules against various species of multidrug-resistant (MDR) and susceptible bacteria, fungi, viruses, susceptible and MDR cancer lines as well as against diabetes, hypertension, and obesity [5–10]. During a pandemic like that of COVID-19, the use of plants by the African population has proven to be lifesaving and has been an effective solution in the management of this disease. However, questions arise about the anarchic use, which supports the idea of the World Health Organization (WHO) on the safety of the use of plants and, consequently, the toxic and undesirable effects it may cause [11]. *Bauhinia thonningii* (Schumach.) Milne-Redh. (Caesalpiniaceae) is a medicinal plant used in the traditional medicine in the treatment of several pathologies such as dysentery, fever, respiratory ailments, snake bites, hookworm, skin diseases, male erectile malfunction, gastro-intestinal track problems, inflammation, bilharzia, eye diseases, malaria, and catarrh [12, 13]. It has been recommended that plants used traditionally against immune and skin disorders, inflammations as well as microbial, parasitic, and viral infections should be screened for cancer drug discovery as these diseases bear reference to cancer or cancer-like symptoms [14, 15]. This is the rationale for investigating the cytotoxic potential of this plant. The study was extended to the isolation of the cytotoxic constituent and the toxicological survey of the leaf’s methanol extract of the plant. Previous studies have shown that this plant possesses several pharmacological activities. In effect, Abdelwahab and collaborators have demonstrated the gastroprotective effects of the methanol and chloroform extracts of the leaves of *Bauhinia thonningii* leaves on ethanol-induced gastric ulceration in Sprague Dawley rats [16]. They showed that the gastroprotective mechanism of the botanical was mediated by the modulation of Periodic acid-Schiff reactive substances, malondialdehyde, and proteomics biomarkers such as creatine kinase, malate dehydrogenase, ATP synthase, actin, and thioredoxin [16]. This plant also displayed hyaluronidase, phospholipase A2 and protease inhibitory activity, indicating the potential against the necrosis-inducing enzymes of snake venom [17]. The leaf extract of this plant displayed antimicrobial activities against *Escherichia coli, Staphylococcus aureus, Enterobacter* species*, Streptococcus pneumoniae, Corynebacterium pyogenes, Enterococcus faecalis, Acinetobacter* species*, and Pseudomonas aeruginosa* [18]. Moreover, the investigation of the antibacterial activity of the plant led to the identification of 6-C-methylquercetin-3,4'-dimethyl ether which displayed prominent inhibitory effects against the resistant phenotypes of Gram-negative bacterium *Klebsiella pneumoniae* and the Gram-positive bacterium *Staphylococcus aureus* [13]. The hypoglycemic and anti-hyperglycemic properties of the plant was also demonstrated in rats [19]. Various leaf extracts of this plant had hypoglycemic effect through their ability to reduce fasting blood glucose in alloxan-induced diabetic rats [20]. The antiviral activity of the leaves of this plant has also been reported against poliovirus, astrovirus, herpes simplex viruses, and parvovirus [21]. Leaf extract of this plant also displayed antioxidant effect through exhibited 2, 2-dipheny l-picrylhydrazyl (DPPH) and (2,2′-azino-bis-(3-ethylbenzothiazolin-6-sulfonic acid) (ABTS) scavenging activities [22].

## Methods

### Plant material and extraction

The leaves of *Bauhinia thonningii* (Schumach.) Milne-Redh. (Caesalpiniaceae) were collected in Bandjoun (5°21′N 10°24′E) in the West region of Cameroon. This plant is not an endangered species, and it was collected in open community field and therefore no prior permission was required. The plant parts (leaves, bark) and the whole plant were identified in the Cameroon National Herbarium (HNC) under the voucher code 33,258/HNC, and furthermore, the plant name *Bauhinia thonningii* (Schumach.) Milne-Redh. (Caesalpiniaceae) was further authenticated using the plant index database, *the World Flora Online,* under the reference code WFO-0001054410. The leaves were dried and powdered, and 2.0 kg were soaked in 15 L of methanol (CH_3_OH) for 48 h. The filtrate obtained using Whatman filter paper grade 1 was evaporated under vacuum to obtain 200.4 g of the crude methanol extract or the botanical (BTL). The botanical was stored at 4 °C for further use.

### Isolation and purification

Part of BTL (194.0 g) was triturated with ethyl acetate (EtOAc) to obtain 96.2 g of EtOAc fraction (BTLa) and 78.3 g of residue (BTLb). The two fractions displayed cytotoxic effects in cancer cell lines and were further subjected to purification. Ninety-six grams (96.0 g) of the BTLa were subjected to column chromatography (CC) using silica gel and eluted with a gradient of *n-*hexane–EtOAc (100:0 to 0:100, v/v) and EtOAc-CH_3_OH (100:0 to 0:100, v/v) to obtain 168 sub-fractions (sub-frs) of 300 mL each collected as follows: *n-*hexane–EtOAc 100:0 (sub-frs 1–24); *n-*hexane–EtOAc 95:5 (sub-frs 25–47); *n-*hexane–EtOAc 85:15 (sub-frs 48–84); *n-*hexane–EtOAc 75:25 (sub-frs 85–108); *n-*hexane–EtOAc 40:60 (sub-frs 109–117); *n-*hexane–EtOAc 25:75 (sub-frs 118–132); EtOAc-CH_3_OH 100:0 (sub-frs 133–144); EtOAc-CH_3_OH 90:10 (sub-frs 145–160), and EtOAc–MeOH 0:100 (sub-frs 161–168). Based on their TLC profiles, these fractions were pooled into seven major sub-fractions (A-G) as follows: sub-fractions A (0% *n-*hexane–EtOAc, sub-frs 1–22, 10.2 g), B (0%-15% *n-*hexane–EtOAc, sub-frs 23–62, 7.5 g), C (15%-25% *n-*hexane–EtOAc, sub-frs 63–85, 8.0 g), D (25%-75% *n-*hexane–EtOAc, sub-frs 86–125, 25.7 g), E (75%-100% *n-*hexane–EtOAc, sub-frs 126–137, 10.5 g), F (0%-10% EtOAc-CH_3_OH, sub-frs 138–155, 5.7 g), and G (10%-100% EtOAc-CH_3_OH, sub-frs 156–168, 6.5 g). Sub-fractions A and B contained mainly fatty acids and were not further investigated. Compounds 4 (15.1 mg) and 9 (19.9 mg) precipitated from fraction C (2.1 g), while compound 10 (25.0 mg) precipitated from fraction E, obtained after simple filtration with EtOAc. Fraction D was subjected to repeated silica gel CC using a gradient elution of *n-*hexane–EtOAc (100:0 to 50:50, v/v) to afford 75 sub-frs of 75 mL each as follows: *n-*hexane–EtOAc 95:5 (sub-frs 1–33), *n-*hexane–EtOAc 85:15 (sub-frs 34–49), *n-*hexane–EtOAc 70:30 (sub-frs 50–63), and *n-*hexane–EtOAc 50:50 (sub-frs 64–75). Based on their TLC profiles, sub-fractions 44–48 and 50–51 were pooled separately and identified as a mixture of compounds, whereas other sub-fractions obtained in a very small amount were not further investigated. The mixture of compounds was treated and purified by CC over Sephadex LH-20 eluting with CH_2_Cl_2_-CH_3_OH (50:50), v/v to yield compound 1 (9.9 mg) from sub-frs 44–48, as well as 2 (12.1 mg) and 8 (6.1 mg) from sub-frs 50–51.

The fraction BTLb (77.0 g) was subjected to repeated silica gel CC using a gradient elution of *n-*hexane–EtOAc (100:0 to 0:100, v/v) and EtOAc-CH_3_OH (100:0 to 10:90, v/v) to afford 67 sub-frs of 200 mL each collected as follows: *n-*hexane–EtOAc 100:0 (sub-frs 1–7), *n-*hexane–EtOAc 85:15 (sub-frs 8–24), *n-*hexane–EtOAc 75:25 (sub-frs 25–30), *n-*hexane–EtOAc 60:40 (sub-frs 31–40), EtOAc-CH_3_OH 100:0 (sub-frs 41–55), and EtOAc-CH_3_OH 90:10 (sub-frs 56–67). Based on their TLC profiles, these sub-frs were pooled together into the five major sub-frs (I-V) as follows: I (0%-15% *n-*hexane–EtOAc, sub-frs 1–23, 8.0 g), II (15%-25% *n-*hexane–EtOAc, sub-frs 24–28, 7.5 g), III (25%-40% *n-*hexane–EtOAc, sub-frs 29–40, 12.0 g), IV (100% EtOAc, sub-frs 41–47, 30.7 g), and V (0%-10% EtOAc-CH_3_OH, sub-frs 48–67, 10.5 g). A precipitate in powder form was observed from fractions II-IV. After simple filtration and purification with ethyl acetate, compounds 7 (8.1 mg), 3 (11.1 mg), and mixture were obtained from fractions II, III, and IV, respectively. The separation of a mixture was possible after CC over Sephadex LH-20 eluting with CH_2_Cl_2_-CH_3_OH (50:50), v/v to afford compounds 5 (15.9 mg) and 6 (17.2 mg).

### Cancer cell lines and cell culture

In this study, a panel of 9 cancer cell lines as well as the normal AML12 hepatocytes were used. The source of these cell lines has been elucidated earlier [23]. The drug-sensitive CCRF-CEM and its multidrug-resistant P-glycoprotein (P-gp)-overexpressing subline, CEM/ADR5000 have been previously reported [24–26]. The sources of the HepG2 hepatocarcinoma [27], U87MG glioblastoma cells and its resistant epidermal growth factor receptor (EGFR) transfected subline, U87MG.*ΔEGFR* [28], HCT116 *p53*^+*/*+^ colon adenocarcinoma cells and its resistant knock-out congener, HCT116 *p53*^*−/−*^ [29], MDA-MB-231-*pcDNA3* breast adenocarcinoma cells and its multidrug-resistant breast cancer resistance protein (BCRP)-transfected MDA-MB-231-*BCRP* clone 23 subline [30] have also been described. To compare the activity of samples in HepG2 cells, the AML12 hepatocytes were used [31]. The cell culture media used included RPMI-1640 for CCRF-CEM cells and CEM/ADR5000 cells as well as DMEM for other (adherent) cells. These culture media were used as complete media with additional 10% fetal bovine serum and 1% penicillin/streptomycin (Invitrogen, Eggenstein, Germany). To maintain their resistance patterns, CEM/ADR5000 cells were cultured in a complete medium with additional 5000 ng/mL doxorubicin whereas MDA-MB-231/*BCRP*, HCT116 *p53*^*−/−*^, and U87MG.*ΔEGFR* cells were cultured in a complete medium with additional geneticin at 800 ng/mL and 400 µg/mL, respectively [32, 33].

### Cytotoxicity evaluation

To determine the cytotoxicity of the botanicals (BTL, BTLa, and BTLb), phytoconstituents (1–10) as well as the positive control, doxorubicin, the resazurin reduction-based assay (RRA) was used as earlier described [34] under experimental conditions applied previously [27, 33]. Botanicals were tested in the concentration range of 0.63–80 µg/mL, the isolated compounds at 0.78—100 µM; the treated cells were incubated for 72 h; the fluorescence was measured at the excitation wavelength of 544 nm and at the emission wavelengths of 590 nm using Infinite M2000 Pro™ plate reader (Tecan, Crailsheim, Germany). The data were expressed as IC_50_ values of tested samples defined as the concentration required to inhibit 50% of the cell proliferation. They were deduced from a calibration curve by linear regression from Microsoft Excel [35]. The degree of resistance (D. R.) of cancer cells towards the investigated samples was determined as the ratio of the IC_50_ value in the resistant cell line *versus* IC_50_ value in the corresponding sensitive cell line [36, 37]; the ratio of the IC_50_ value of the normal AML12 hepatocytes *versus* that of HepG2 cells was referred to as the selectivity index (S.I.) [38].

### Analysis of cell cycle distribution and apoptosis

The distribution of the cell cycle in CCRF-CEM cells as a result of the application of the botanical BTL, compound 8, doxorubicin, or solvent control, dimethyl sulfoxide (DMSO) was evaluated by the combined flow cytometry and propidium iodide staining similarly to the previously reported experimental conditions [39]. The concentrations of samples used to treat the CCRF-CEM cells were 0.25, 0.5, 1, and 2 folds the IC_50_. The treated cells (1 mL; 1 × 10^6^ cells) were incubated for 24 h at 37 °C, 5% CO_2_ and 95% relative humidity referred to as the standard cell culture condition. The cell cycle analysis was further monitored by means of the BD Accury C6 Flow Cytometer (BD Biosciences, Heidelberg, Germany) [39, 40]. Assays were performed separately at least three times in triplicates.

### Analysis of apoptosis by the combined annexin V/propidium iodide staining and flow cytometry

To detect the apoptotic CCRF-CEM cells resulting from the application of BTL, compound 8, doxorubicin, or DMSO, the annexin V/propidium iodide (PI) staining combined with the flow cytometry was used in similar experimental conditions as reported earlier [33, 41–43]. The concentration of samples used and CCRF-CEM cells were those as reported above under the standard cell culture conditions. To detect the apoptotic cells, the fluorescein isothiocyanate (FITC)-conjugated annexin V/PI assay kit (eBioscience™ Annexin V; Invitrogen, San Diego, USA) was applied to treated cells after 24 h incubation followed by the measurements with BD Accury C6 Flow Cytometer (BD Biosciences) [42, 43]. The assays were performed separately three times and in triplicate.

### Caspases activity evaluation by the combined Caspase-Glo assay and spectrophotometry

To detect the activity of caspases in CCRF-CEM cells resulting from the application of BTL and compound 8, the Caspase-Glo assay combined with the spectrophotometric determination of their luminescence was applied in similar experimental conditions as reported earlier [44]. The concentrations of samples used to treat the CCRF-CEM cells were 0.25, 0.5, 1, and 2 folds the IC_50_. The amount of the treated cells was 1.5 × 10^4^ cells for the caspase 3/7 assay or 3 × 10^4^ cells for the caspase 8 and caspase 9 assays in 100 µL. The treated cells were then incubated for 6 h under the standard cell culture conditions as described above. Subsequently, the activity of caspases was detected by a spectrophotometry measurement of luminescence using 100 µL caspase solution from the Caspase-Glo 3/7, 8, and 9 Assay kits (Promega, Mannheim, Germany) with an Infinite M2000 ProTM plate reader (Tecan).

### Assessment of MMP alteration and ROS production

To quantify the modification of the mitochondrial membrane potential (MMP) and the reactive oxygen species (ROS) production in CCRF-CEM cells resulting from the application of BTL, either compound 8 or DMSO (negative control), or the respective positive controls for MMP or ROS evaluations, valinomycin or H_2_O_2_ (Sigma-Aldrich, Taufkirchen, Germany), the 5,5′,6,6′-tetrachloro-1,1′,3,3′-tetraethylbenzimidazolylcarbocyanine iodide (JC-1; Biomol, Hamburg, Germany) staining and the 2´,7´-dichlorodihydrofluorescein diacetate (H_2_DCFH-DA) (Sigma-Aldrich) staining, respectively for MMP and ROS measurements, were combined with the flow cytometry used in similar experimental conditions, as earlier reported [45]. The concentrations of samples used to treat the CCRF-CEM cells were 0.25, 0.5, 1, and 2 folds the IC_50_. The treated cells (1 mL; 1 × 10^6^ cells) were incubated for 24 h under the standard cell culture condition. Cells were further stained for 30 min with 10 µL of staining solution (JC-1 for ROS evaluation or H_2_DCFH-DA solution for ROS evaluation) according to the manufacturer’s protocol. Using the LSRFortessa FACS analyzer (Becton–Dickinson, Heidelberg, Germany), the amount of 1 × 10^4^ cells was further measured as described earlier [31, 45–47].

### Animal experimentation in toxicological studies

Thirty-eight *Wistar* albino rats at 8 weeks of age weighting between 160 and 200 g were investigated. The animal breeding took place in the animal house, University of Dschang, Cameroon. The animals were placed individually in polypropylene cages at 25 ± 1 ºC in a 12 h: 12 h dark: light cycle. Food and tap water were given to them ad libitum. The animals were treated according to the protocol for the care and use of laboratory animals based on the ethical considerations of the Institutional Ethical Committee of the University of Dschang, Cameroon (Project No. BCH1202/FS/UDS/2020).

### Acute oral toxicity of BTL

The acute toxicity study was conducted according to the OECD guideline with six healthy female rats (age: 8 weeks; mass: 150–155 g). They were nulliparous [48] and were randomly divided into two groups of three rats per group. Group I was the control group whereas Group II received a dose of 5000 mg/kg of BTL one after another and in a time interval of 48 h. Each animal received the extract by gavage using an endogastric tube. The trial ended when the three serial rats survived to a limited dose of 5000 mg/kg. After the administration of the test extract (BTL), mortality and clinical signs were noted for the first 3 h and thereafter for 14 days of drug administration [49]. After 14 days of observation, animals were anesthetized with diazepam/ketamine (0.2/0.1 mL per 100 g of the animal) association and autopsied. Eventual macroscopic changes were observed in organs such as the liver, kidneys, lungs, and heart.

### Sub-chronic oral toxicity study of BTL

The sub-chronic oral toxicity study was conducted according to the standard methods [50] with thirty-two (32) rats (16 males and 16 females; age: 8 weeks; mass: 180–200 g) randomly divided into 4 groups of 4 rats per group and sex. The rats received BTL at 250 mg/kg, 500 mg/kg, and 1000 mg/kg doses of body weight (b. w.) by endogastric gavage for 28 days in batches 1, 2, and 3, respectively. Batch 4 was the control group for each sex. Food consumption and body weight were determined every four days, as previously described [51]. On day 29 of the test, after gavages, the animals were subjected to a 12 h food fast, at the end of which urine was collected. Subsequently, the animals were sacrificed and autopsied. The sacrifice was done as follows: the rats were anesthetized with intra-peritoneal injections of diazepam (0.1 mL/100 g of the animal) and ketamine (0.2 mL/100 g of the animal). The blood was collected by cardiac puncture and then first introduced into a tube containing an anticoagulant, ethylenediaminetetraacetic acid (EDTA), for the hematological analysis using QBC Autoread Plus (Ipswich, United Kingdom) and, secondly, into a tube without anticoagulant for the preparation of the serum. The serum obtained was used to determine biochemical parameters such as creatinine, urea, aspartate aminotransferase (AST), alanine aminotransferase (ALT), total cholesterol, high-density lipoprotein (HDL), low-density lipoprotein (LDL), triglycerides, and total proteins using commercial SPINREACT brand kits (Barcelona, Spain). After that, certain organs such as the heart, kidneys, liver, lung, and spleen were immediately removed. These organs were used to determine the relative weight of the organs. One part of each organ for histopathological analysis was stored in 10% buffered formalin, as described earlier [52].

### Statistical analysis

Statistical analyses were performed using SPSS version 23.0 for Windows. The results were expressed as mean value ± standard deviation (SD), and the comparisons were performed by the analysis of variance using a one-way analysis of variance test. Differences between the averages of control and drug-treated groups where they existed were separated using the Waller–Duncan test. *P-*values less than 0.05 were considered statistically significant.

## Results

### Phytochemistry

**Fig. 1:**
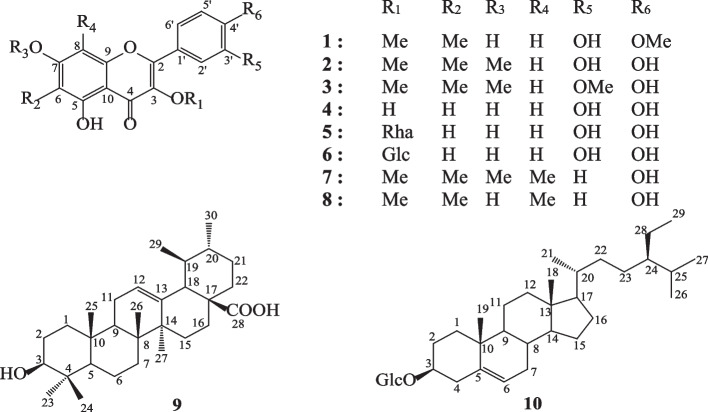
Chemical structures of compounds isolated from the leaves of *Bauhinia thonningii*. 6-C-methylquercetin-3, 4'-dimethyl ether (1); 6-C-methylquercetin-3,7-dimethyl ether (2); 6-C-methylquercetin 3,7,3'-trimethyl ether (3); quercetin (4); quercetin-3-*O*-_L_-rhamnopyranoside (5); quercetin-3-*O-β*-glucopyranoside (6); 6,8-C-dimethylkaempferol 3,7-dimethyl ether (7); 6,8-C-dimethylkaempferol-3-methyl ether (8); ursolic acid (9); 3-*O*-*β*-_D_-glucopyranoside of *β*-sitosterol (10)

The phytochemical study of *Bauhinia thonningii* led to the isolation and identification of 10 compounds. Their chemical structures are shown in Fig. 1. The general experimental procedure is provided in Supplementary Information S1 whereas their spectral properties including ^1^H NMR, ^13^C NMR, and HMBC data are shown in Supplementary Information S2. The phytochemicals were identical to those previously described in the literature. They were six flavonols: 6-C-methylquercetin-3, 4'-dimethyl ether C_18_H_16_O_7_ (1; yellow powder; *m/z* 345.09; m. p. 195–197 °C) [13], 6-C-methylquercetin-3,7-dimethyl ether C_18_H_16_O_7_ (2; yellow powder; *m/z* 344.32; m. p. 195–197 °C) [13, 53], 6-C-methylquercetin 3,7,3'-trimethyl ether C_19_H_18_O_7_ (3; yellow powder; *m/z*344.32; m. p. 185–187 °C) [13, 53], quercetin C_15_H_10_O_7_ (4; yellow powder; *m/z* 302.24; m. p. 314–317 °C) [53], 6,8-C-dimethylkaempferol 3,7-dimethyl ether C_19_H_18_O_6_ (7; yellow powder; *m/z* 342.35; m. p. 285–287 °C) [13, 53], and 6,8-C-dimethylkaempferol-3-methyl ether C_18_H_16_O_6_ (8; yellow powder; *m/z* 228.09; m. p. 250–253 °C) [13, 53]; two flavonol glycosides: quercetin-3-*O*-_L_-rhamnopyranoside C_21_H_20_O_11_ (5; yellow powder; *m/z* 448.10; m. p. 180–182 °C)[13, 53], and quercetin-3-*O*-*β*-glucopyranoside C_21_H_20_O_12_ (6; yellow powder; *m/z* 464.38; m. p. 176–179 °C) [13, 53], one triterpenoids ursolic acid C_30_H_48_O_3_ (9; white powder; *m / z* 456.71; m. p. 283–285 °C)[13, 54], and one sterol glycoside, 3-*O*-*β*-_D_-glucopyranoside of *β*-sitosterol C_35_H_60_O_6_ (10; white powder; *m / z* 456.71; m. p. > 212 °C) [13, 55]. These compounds were tested alongside the botanicals towards a panel of human cancer cell lines, and the results are shown in Table 1.

**Table 1:** Cytotoxicity of the methanol extract (BTL), fractions (BTLa and BTLb) from the leaves of *Bauhinia thonningii* as well as doxorubicin in the studied cancer cells

Cell lines	Samples, IC_50_ values in µg/mL or µM (for Doxorubicin), degrees of resistance^a^ or selectivity index^b^ (in bracket)			
	**Methanol extract**	**Fractions**	**Positive control**	
	**BTL**	**BTLa**	**BTLb**	**Doxorubicin**
CCRF-CEM	**18.13 ± 1.42**	**19.51 ± 0.96**	**17.05 ± 1.03**	**0.02 ± 0.00**
CEM/ADR5000 Degree of resistance^a^	29.20 ± 1.77 (1.61)	**19.16 ± 1.68** (0.98)	23.49 ± 2.31 (1.37)	122.96 ± 10.94 (6,683.00)
MDA-MB-231-*pcDNA*	23.09 ± 2.19	**17.76 ± 2.01**	24.76 ± 1.93	**0.13 ± 0.01**
MDA-MB-231-*BCRP* Degree of resistance	26.22 ± 1.61 (1.14)	20.08 ± 0.89 (1.13)	64.74 ± 3.82 (2.61)	**0.79 ± 0.08** (6.14)
HCT116 *p53*^+*/*+^	40.84 ± 3.12	43.60 ± 2.55	52.33 ± 3.71	**0.48 ± 0.06**
HCT116 *p53*^*−/−*^ Degree of resistance	28.14 ± 1.83 (0.70)	47.40 ± 3.28 (1.08)	48.91 ± 2.89 (0.93)	**1.78 ± 0.08** (3.73)
U87MG	22.66 ± 1.76	**12.46 ± 0.67**	**16.55 ± 1.40**	**0.26 ± 0.03**
U87MG.Δ*EGFR* Degree of resistance	24.43 ± 0.89 (1.07)	**15.61 ± 1.72** (1.25)	29.05 ± 1.99 (1.21)	**0.98 ± 0.07** (3.79)
HepG2	24.38 ± 1.26	**18.30 ± 0.77**	30.65 ± 2.16	**4.56 ± 0.48**
AML12 Selectivity index^b^	70.12 ± 3.25 (2.87)	> 80 (4.37)	> 80 (2.61)	> 50 (> 11.59)

^a^The degree of resistance was determined as the ratio of IC_50_ value in the resistant divided by IC_50_ in the sensitive cell line; CEM/ADR5000 *versus* CCRF-CEM, MDA-MB-231-*BCRP versus* MDA-MB-231-*pcDNA,* HCT116 *p53*^*−/−*^* versus* HCT116 *p53*^+*/*+^ and U87.MGΔEGFR *versus* U87.MG; ^b^The selectivity index: ratio of IC_50_ in AML12 *versus* IC_50_ in HepG2 cells [36, 37]. Tested samples were the methanol extract from leaves (BTL), ethyl acetate fraction from BTL (BTLa), and residue obtained after partition of BTL with ethyl acetate (BTLb) of *Bauhinia thonningii*. In bold: significant cytotoxic effect [61, 63]

### Cytotoxicity of the studied samples

The data on the cytotoxicity of BTL, BTLa, BTLb, and doxorubicin are displayed in Tables 2 and 3. The three botanicals including the crude extract BTL as well as the fractions BTLa and BTLb had cytotoxic effects towards the nine tested cancer cell lines (Table 1). The obtained IC_50_ values varied from 18.13 µg/mL (against CCRF-CEM cells) to 40.84 µg/mL (against HCT116 *p53*^+*/*+^ cells) for BTL, from 12.46 µg/mL (against U87MG cells) to 47.40 µg/mL (against HCT116 *p53*^*−/−*^cells) for BTLa, and from 16.55 µg/mL (against U87MG cells) to 64.74 µg/mL (against MDA-MB-231-*BCRP* cells) for BTLb. The obtained IC_50_ values with doxorubicin varied 0.02 µM (against CCRF-CEM cells) to 122.96 µM (against CEM/ADR5000 cells) (Table 1). The purification of BTLa led to compounds 1, 2, 4, and 8 whereas that of BTLb led to compounds 3, 5, 6, and 7. The cytotoxicity of compounds was further determined in the nine cancer cell lines, as for the botanicals, and the results are summarized in Table 2. Compounds 1–3 and 5–8 had antiproliferative effects with the IC_50_ values below 50 µM against all the investigated cancer cell lines whilst compounds 4 and 10 were selectively active. No activity at up to 50 µM was detected with compound 9. The IC_50_ values below 10 µM were obtained with compounds 2, 5, 6, 7, and 8 toward 3, 3, 3, 5, and 7 of the 9 tested cancer cell lines, respectively (Table 2). The best cytotoxic effects with IC_50_ values below 5 µM were achieved with compounds 7 against CEM/ADR5000 leukemia cells (2.86 µM) and MDA-MB-231-*pcDNA* breast adenocarcinoma cells (1.93 µM) and as well as compound 8 against CCRF-CEM leukemia cells (3.03 µM), CEM/ADR5000 cells (2.42 µM), MDA-MB-231-*pcDNA* (2.34 µM), and HCT116 *p53*^*−/−*^ cells (3.41 µM) (Table 2). Amongst these most active compounds, compounds 2, 6, and 8 as well as the botanicals were more selective to normal AML12 hepatocytes compared to HepG2 hepatocarcinoma cells, with selective indexes above 2 (Tables 1 and 2). Regarding the best samples, normal sensitivity or hypersensitivity (collateral sensitivity) of CEM/ADR5000 cells *versus* CCRF-CEM cells was achieved *vis-à-vis* BTLa (D. R. of 0.98), compounds 2 (D. R. of 0.76), 5 (D. R. of 0.83), 6 (D. R. of 0.90), 7 (D. R. of 0.43), and 8 (D. R. of 0.80). This was also the case for MDA-MB-231-*BCRP* cells *versus* MDA-MB-231-*pcDNA* cells *vis-à-vis* BTL (D. R. of 1.14) BTL a (D. R. of 1.13) and compound 3 (D. R. of 1.20), HCT116 *p53*^*−/−*^ cells *versus* HCT116 *p53*^+*/*+^ cells *vis-à-vis* BTL (D. R. of 0.70), BTLa (D. R. of 1.08), BTLb (D. R. of 0.93), compounds 2 (D. R. of 0.75), 5 (D. R. of 0.73), 6 (D. R. of 0.57), 7 (D. R. of 0.50), and 8 (D. R. of 0.36), and U87.MGΔ*EGFR* cells *versus* U87.MG cells *vis-à-vis* BTL (D. R. of 1.07), compounds 2 (D. R. of 0.85), and 7 (D. R. of 0.93) (Table 2).

**Table 2:** Cytotoxicity of the phytoconstituents isolated from the leaves of *Bauhinia thonningii* against the studied cancer cells

Cell lines	Samples, IC_50_ (µM), degrees of resistance^a^ or selectivity index^b^ (in bracket)									
	**1**	**2**	**3**	**4**	**5**	**6**	**7**	**8**	**9**	**10**
CCRF-CEM	14.23 ± 0.94	12.02 ± 1.25	17.85 ± 1.67	27.32 ± 3.12	**8.73 ± 0.62**	**9.12 ± 0.59**	**6.61 ± 0.41**	**3.03 ± 0.37**	> 50	44.28 ± 3.91
CEM/ADR5000 Degree of resistance^a^	10.28 ± 0.44 (0.72)	**9.11 ± 0.18** (0.76)	10.24 ± 0.30 (0.57)	> 50 (> 1.83)	**7.21 ± 0.61** 0.83)	**7.88 ± 0.58** (0.90)	**2.86 ± 0.17** (0.43)	**2.42 ± 0.30** (0.80)	> 50	> 50 (> 1.13)
MDA-MB-231-*pcDNA*	11.28 ± 1.04	**9.84 ± 0.66**	11.02 ± 0.58	42.28 ± 3.29	**7.24 ± 0.47**	**6.99 ± 0.28**	**1.93 ± 0.19**	**2.34 ± 0.31**	> 50	> 50
MDA-MB-231-*BCRP* Degree of resistance	16.98 ± 2.05 (1.51)	14.29 ± 1.12 (1.45)	13.19 ± 1.48 (1.20)	> 50 (> 1.18)	16.82 ± 1.26 (2.32)	12.30 ± 0.97 (1.76)	**8.15 ± 0.73** (4.22)	**8.26 ± 0.66** (3.53)	> 50	> 50
HCT116 *p53*^+*/*+^	21.18 ± 2.45	12.27 ± 1.24	19.78 ± 0.78	> 50	17.93 ± 1.26	21.84 ± 2.11	19.50 ± 2.05	**9.59 ± 0.84**	> 50	> 50
HCT116 *p53*^*−/−*^ Degree of resistance	10.77 ± 1.11 (0.51)	**9.20 ± 0.45** (0.75)	17.23 ± 0.83 (0.87)	> 50	13.20 ± 1.14 (0.73)	12.49 ± 1.36 (0.57)	**9.71 ± 0.76** (0.50)	**3.41 ± 0.35** (0.36)	> 50	> 50
U87MG	27.15 ± 3.07	24.78 ± 2.22	31.02 ± 2.65	> 50	23.65 ± 1.77	19.03 ± 2.01	19.27 ± 1.62	**8.01** ± 0.84	> 50	> 50
U87MG.Δ*EGFR* Degree of resistance	26.63 ± 1.13 (0.98)	21.09 ± 2.40 (0.85)	19.75 ± 0.96 (0.64)	> 50	31.09 ± 1.85 (1.31)	33.54 ± 2.79 (1.75)	18.01 ± 2.21 (0.93)	15.38 ± 0.64 (1.92)	> 50	> 50
HepG2	23.46 ± 0.59	19.55 ± 1.44	23.57 ± 3.23	> 50	28.29 ± 1.74	21.48 ± 2.25	30.67 ± 1.86	19.39 ± 2.07	> 50	> 50
AML12 Selectivity index^b^	> 50 (> 2.13)	> 50 (> 2.56)	> 50 (> 212)	> 50	> 50 (> 1.77)	> 50 (> 2.33)	> 50 (> 1.63)	> 50 (> 2.58)	> 50	> 50

In bold: significant cytotoxic effect [61, 63]; Compounds: 6-C-methylquercetin-3, 4'-dimethyl ether (1); 6-C-methylquercetin-3,7-dimethyl ether (2); 6-C-methylquercetin 3,7,3'-trimethyl ether (3); quercetin (4); quercetin-3-*O*-_L_-rhamnopyranoside (5); quercetin-3-O-β-glucopyranoside (6); 6,8-C-dimethylkaempferol 3,7-dimethyl ether (7); 6,8-C-dimethylkaempferol-3-methyl ether (8) ursolic acid (9) and 3-*O*-*β*-_D_-glucopyranoside of *β*-sitosterol (10); ^a^ The degree of resistance was determined as the ratio of the IC_50_ in the resistant divided by the IC_50_ in the sensitive cell lines; CEM/ADR5000 *versus* CCRF-CEM, MDA-MB-231-*BCRP versus* MDA-MB-231-*pcDNA,* HCT116 *p53*^*−/−*^* versus* HCT116 *p53*^+*/*+^ and U87.MGΔEGFR *versus* U87.MG; ^b^ The selectivity index: ratio of IC_50_ in AML12 *versus* IC_50_ in HepG2 cells [36, 37]

**Table 3:** Effect of BTL on hematological parameters of animals

Sexes	Studies animals, doses, and values of investigated parameters				
	**Parameters**	**Doses of BTL and values of parameters**			
		**Control (0 mg/kg)**	**250 mg/kg**	**500 mg/kg**	**1000 mg/kg**
Females	WBC (10^3^/ml)	8.68 ± 1.79^a^	7.96 ± 0.89^a^	7.50 ± 1.62^a^	7.60 ± 1.41^a^
	RBC (10^6^/mL)	7.92 ± 0.19^a^	8.09 ± 0.27^a^	8.34 ± 0.20^a^	8.07 ± 0.24^a^
	Hb (g/dL)	13.87 ± 0.33^a^	13.48 ± 0.73^a^	14.23 ± 0.26^a^	14.50 ± 0.37^a^
	HCT (%)	40.52 ± 1.47^a^	41.65 ± 0.21^a^	41.86 ± 0.66^ab^	41.87 ± 1.47^ab^
	PLT (10^3^/mL)	600.83 ± 11.30^a^	588.16 ± 16.57^a^	599.66 ± 11.94^a^	602.00 ± 14.13^a^
	LYM (%)	58.85 ± 4.84^a^	60.18 ± 0.59^a^	54.00 ± 6.64^a^	56.35 ± 3.17^a^
	MID (%)	8.75 ± 0.73^a^	8.00 ± 1.97^a^	7.20 ± 1.13^a^	8.05 ± 1.40^a^
	GRAN%	31.82 ± 4.68^a^	33.38 ± 0.82^a^	34.12 ± 1.81^a^	32.97 ± 5.98^a^
Males	WBC (10^3^/ml)	9.92 ± 0.47^a^	9.30 ± 4.08^a^	8.86 ± 4.10^a^	10.50 ± 1.98^a^
	RBC (10^6^/mL)	8.44 ± 0.61^a^	8.08 ± 0.41^a^	8.33 ± 0.28^a^	7.94 ± 0.70^a^
	Hb (g/dL)	14.66 ± 0.68^a^	13.85 ± 0.36^a^	14.84 ± 0.44^a^	13.47 ± 1.55^a^
	HCT (%)	42.12 ± 1.91^a^	40.15 ± 1.80^a^	42.62 ± 2.10^a^	40.53 ± 2.18^a^
	PLT (10^3^/mL)	667.75 ± 82.77^ab^	568.75 ± 49.64^a^	704.25 ± 98.58^b^	632.00 ± 19.20^ab^
	LYM (%)	53.37 ± 3.01^a^	54.48 ± 3.70^a^	54.77 ± 5.61^a^	55.83 ± 1.65^a^
	MID (%)	9.45 ± 0.50^a^	9.55 ± 1.72^a^	9.22 ± 0.98^a^	10.95 ± 1.49^a^
	GRAN%	32.53 ± 2.52^a^	34.05 ± 2.18^a^	32.11 ± 1.65^a^	32.88 ± 3.05^a^

Data are expressed as mean ± SD, *n* = 4. Values for a given group in a line followed by a different letter as a superscript are significantly different (*P* < 0.05). *RBC* red blood cells, *Hb* hemoglobin, *LYM* lymphocytes, *MID* mean corpuscular volume, *GRAN* granulocytes, *WBC* white blood cells, *PLT* platelets, *HCT* hematocrit

### Cell cycle distribution and apoptosis

**Fig. 2:**
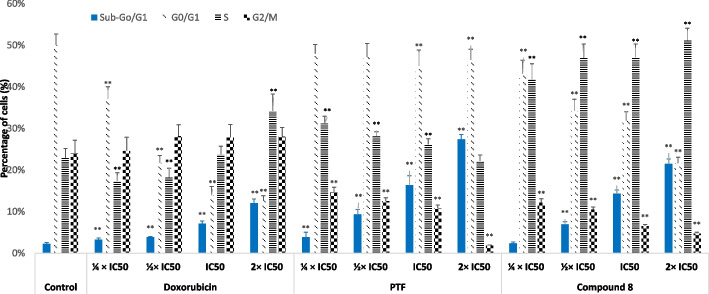
Effects of the crude extract (BTL) and compound 8 from the leaves of *Bauhinia thonningii*, and doxorubicin on the cycle distribution in CCRF-CEM cells after 24 h treatment. IC50 values 14.23 µg/mL for BTL, 3.03 µM for compound 8 and 0.02 µM for doxorubicin. Control cells were treated with DMSO to a final concentration of 0.1%. (**): values were significantly different to those of untreated control (*p* < 0.05); The propidium iodide (PI) staining was used for the cell cycle analysis by the flow cytometry; BTL, compound 8 and doxorubicin induced apoptosis in CCRF-CEM cells as shown with increase in cells in sub-G0/G1 phase. At higher concentration (2 × IC50), BTL induces cell cycle arrest in G0/G1 phase; compound 8 induced cell cycle arrest in G2/M phase; doxorubicin induced cell cycle arrest in G2/M and S phases

**Fig. 3:**
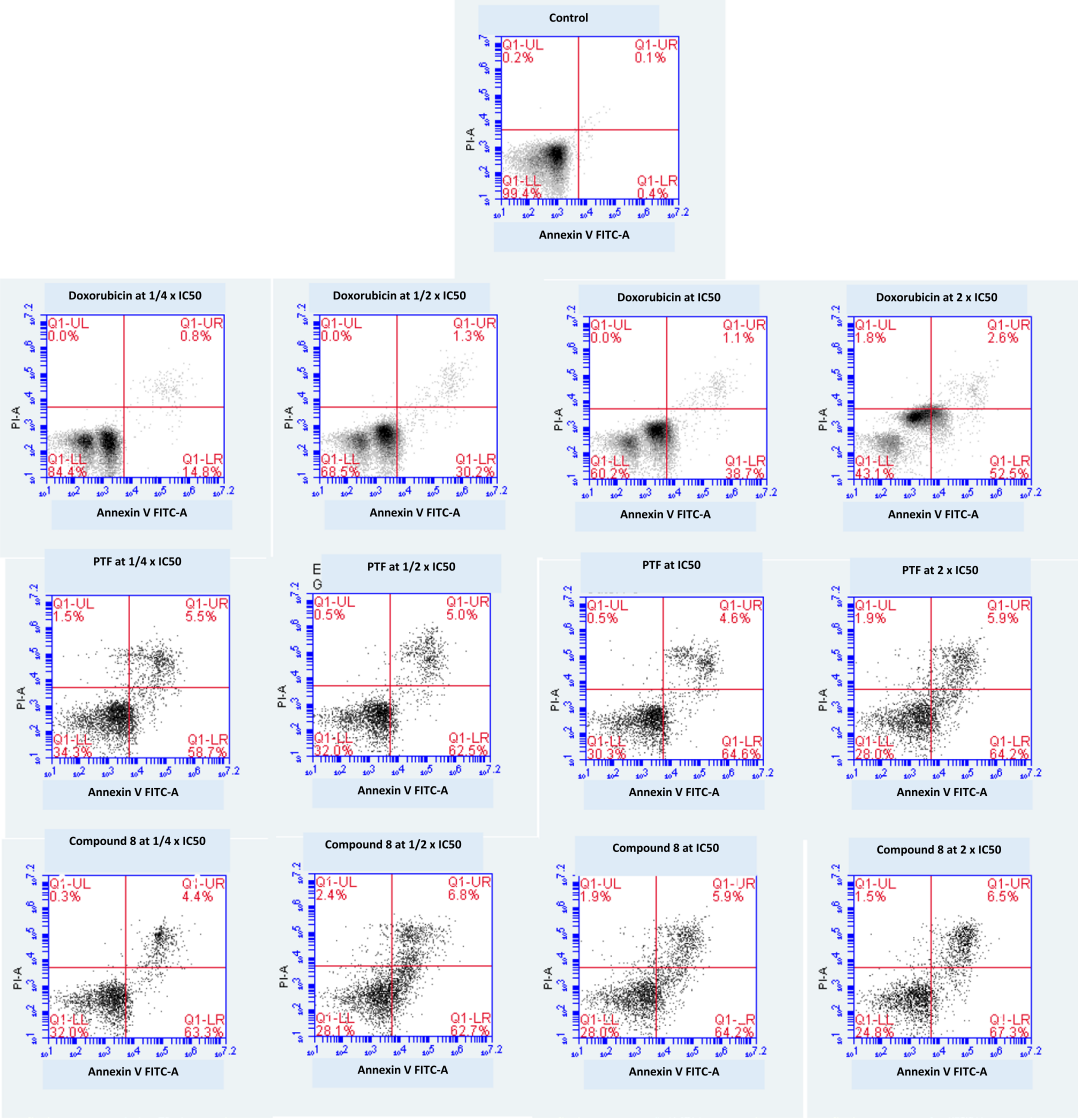
Effects of the crude extract (BTL) and compound 8 from the leaves of *Bauhinia thonningii*, and doxorubicin on apoptosis in CCRF-CEM cells after 24 h treatment. Q1-LL: viable cells with annexin V (-)/PI (-) stain; Q1-LR: early apoptotic cells with annexin ( +)/PI (-) stain; Q1-UR and Q1-UL: late apoptotic cells or necrotic cells with annexin V ( +)/PI ( +) or annexin V (-)/PI ( +) stains. IC_50_ values 14.23 µg/mL for BTL, 3.03 µM for compound 8, and 0.02 µM for doxorubicin. Control cells were treated with DMSO to a final concentration of 0.1%. The annexin V/propidium iodide (PI) staining combined with flow cytometry to detect the apoptosis; BTL, compound 8, and doxorubicin induced early apoptosis with a high proportion of annexin V ( +)/PI (-) cells

The modification of the life cycle in CCRF-CEM after the application of BTL, compound 8, and doxorubicin is shown in Fig. 2. Dose-dependent modifications of the various phases of the cell cycles can be observed because of the treatment with all studied samples. In effect, at higher 2 × IC_50_, BTL induces cell cycle arrest in the G0/G1 phase; compound 8 induces cell cycle arrest in the G2/M phase while doxorubicin induces cell cycle arrest in the G2/M and S phases. Furthermore, the number of cells in the sub-G0/G1 phase also increased significantly (*p* < 0.05) in a concentration-dependent manner upon treatments; within the concentrations ranges of 1/4 × IC_50_ and 2 × IC_50_, the number of cells in the sub-G0/G1 phase varied from 3.92 to 27.40% for BTL, 2.36 to 21.51% for compound 8, and from 3.28 to 12.05% for doxorubicin (Fig. 2). This is an indication that these samples induced apoptosis in CCRF-CEM cells. This was confirmed with annexin V/PI staining as summarized in Fig. 3. BTL, compound 8, and doxorubicin induced early apoptosis with high proportions of annexin V ( +)/PI (-) cells, up to 64.2%, 67.3%, and 52.5% respectively, against 0.4% for non-treated cells.

### Activation of caspases

**Fig. 4:**
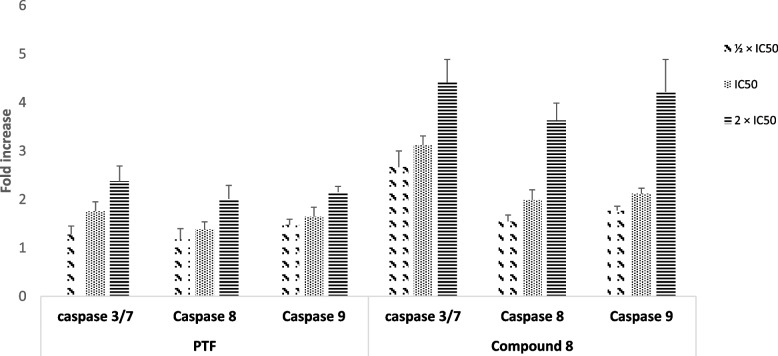
Effects of the crude extract (BTL) and compound 8 from the leaves of *Bauhinia thonningii* on the activity of caspases in CCRF-CEM cells after 6 h treatment. IC_50_ values 14.23 µg/mL for BTL and 3.03 µM for compound 8. Control cells were treated with DMSO to a final concentration of 0.1%. The Caspase-Glo assay was used to detect the activity of Caspases; Caspase activity was expressed as percentage (%) compared to untreated cells. Results display mean values ± SD of three assays. BTL slightly induced the activation of caspases 3/7, 8, and 9; compound 8 induced higher activation of caspases 3/7, 8, and 9

The botanical BTL and the phytochemical 8 were used to treat CCRF-CEM cells at various concentrations for 6 h followed by the determination of caspase activity. The data are depicted in Fig. 4. The activity of caspases 3/7, 8, and 9 in CCRF-CEM cells upon treatments with BTL and compound 8 increased by 2.38-fold, 2.01-fold, and 2.15-fold as well as by 4.41-fold, 3.63-fold, and 4.22-fold, respectively. This clearly suggests the activation of these enzymes.

### States of MMP and ROS

**Fig. 5:**
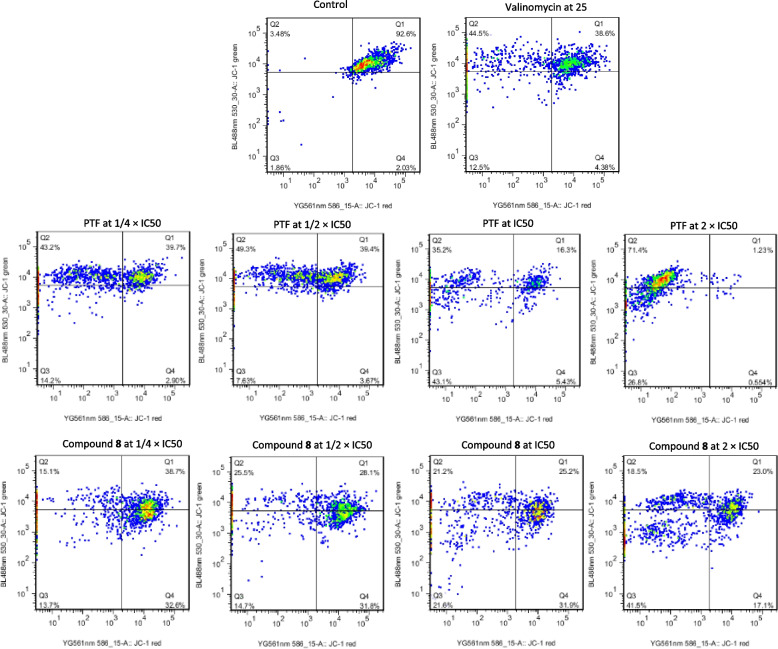
Effects of the crude extract (BTL) and compound 8 from the leaves of *Bauhinia thonningii*, valinomycin on the mitochondrial membrane potential (MMP) of CCRF-CEM cells after 24 h treatment. IC50 values 14.23 µg/mL for BTL and 3.03 µM for compound 8. Control cells were treated with DMSO to a final concentration of 0.1%. The 5,5′,6,6′-tetrachloro-1,1′,3,3′-tetraethylbenzimidazolylcarbocyanine iodide (JC-1) staining combined to flow cytometry was used to analyze the MMP. Q1 represents cells with undamaged MMP, Q2 represents cells with MMP loss whereas Q3-Q4 represents cells with disrupted MMP. BTL, compound 8 or valinomycin strongly altered the integrity of the MMP

**Fig. 6:**
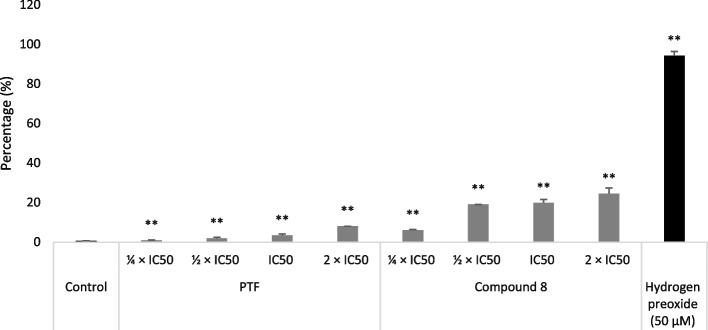
Effects of the crude extract (BTL) and compound 8 from the leaves of *Bauhinia thonningii* and of hydrogen peroxide (H_2_O_2_) on the reactive oxygen species (ROS) production in CCRF-CEM cells upon 24 h treatment. IC_50_ values 14.23 µg/mL for BTL and 3.03 µM for compound 8. Control cells were treated with DMSO to a final concentration of 0.1%. The ROS level was determined by the combined 2´,7´-dichlorodihydrofluorescein diacetate staining and flow cytometry; (**): values are significantly different to those of untreated control (*p* < 0.05). Results display mean values ± SD of three assays. BTL slightly enhanced ROS production; compound 8 enhanced higher ROS production

The state of MMP of CCRF-CEM cells upon application of BTL, compound 8, and valinomycin for 24 h followed by JC-1 staining is shown in Fig. 5. It can be noticed that the MMP in the cells was considerably altered. At 2 × IC_50_, only 1.23%, 23.0%, and 38.6% cells with intact MMP were observed after the application of BTL, compound 8, and valinomycin, respectively. In comparison, up to 92.6% of cells with healthy MMP were recorded in untreated control under the same experimental condition. Concerning ROS levels, a significant dose-dependent increase (*p* < 0.05) of the production in CCRF-CEM cells was recorded upon treatment with BTL, compound 8, and H_2_O_2_ (Fig. 6). For instance, the ROS level in untreated cells was only 0.6% against 8.00%, 24.50% at 2 × IC_50_ in cells treated with BTL and compound 8, respectively, and 94.30% in cells treated with H_2_O_2_ at 50 µM.

### Acute oral toxicity study

The botanical, BTL at dose 5000 mg/kg b. w. in acute administration did not cause deaths, signs of toxicity or mobility, somnolence, salivation, response to noise, stool appearance, etc. in the animals during the first 3 h after administration of the extract and during the 14 days of observation. No macroscopic or microscopic change was observed in organ tissues of animals given 5000 mg/kg b. w. of extract after 14 days of observation (data not shown).

### Sub-chronic oral toxicity study

#### Effect of oral administration of BTL on the evolution of food consumption of rats and body weight

**Fig. 7:**
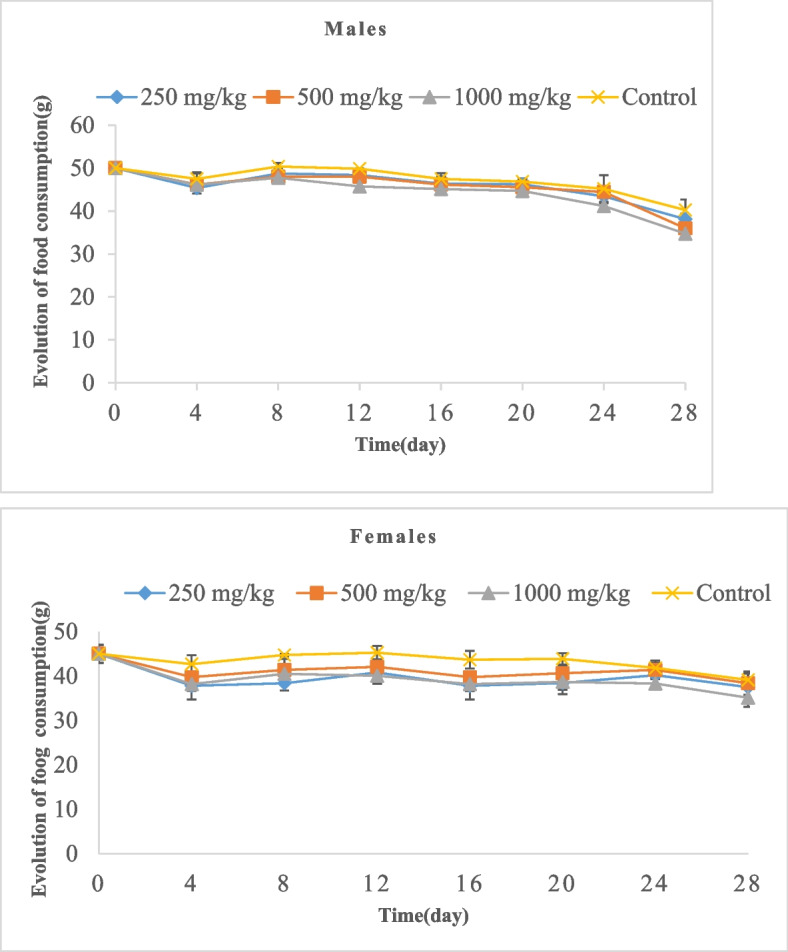
Effect of sub-chronic administration of BTL on food consumption of rats. Data are expressed as mean ± SD, *n* = 4. No significant difference was observed (*p* < 0.05)

**Fig. 8:**
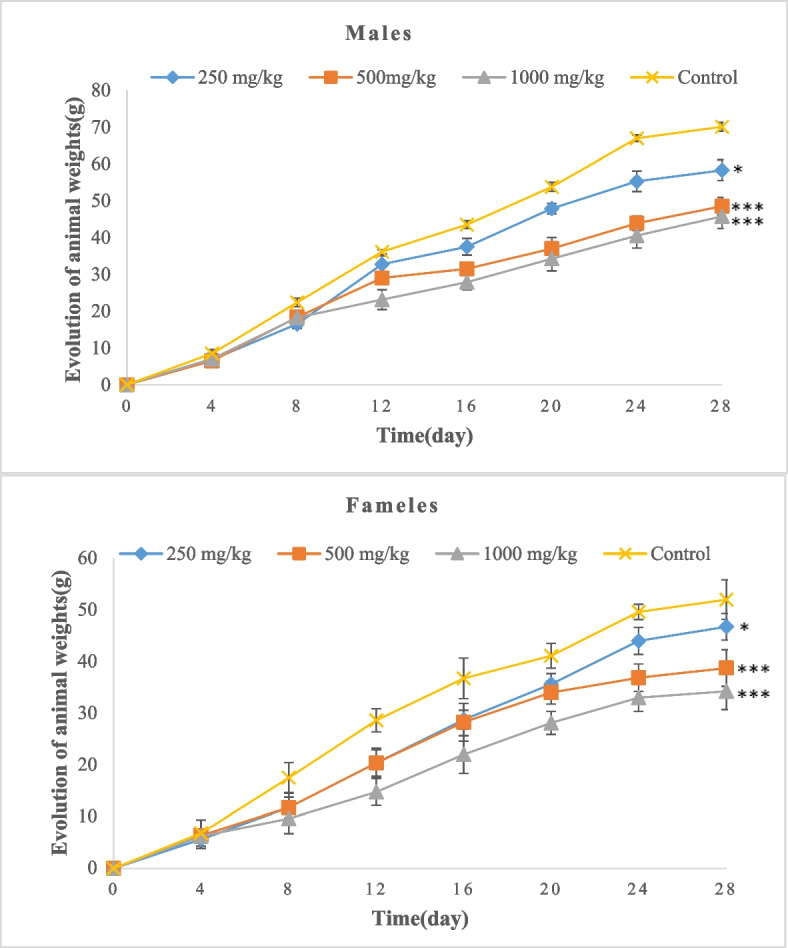
Effect of sub-chronic administration of BTL leaves on body weight of rats. Data are expressed as mean ± SD, *n* = 4. Values for a given group in a line followed by different symbols are significantly different (*p* < 0.05)

Figures 7 and 8 show the evolution of food consumption and body weight of the animals during the 28 days of sub-chronic administration of BTL. The repeated administration of different doses of the extract did not produce a significant difference in the food consumption of male and female rats compared to the control animals (Fig. 7). However, Fig. 8 shows a progressive decrease in body weight in the animals given different doses of extract compared to the control animals. This decrease in body weight was inversely proportional to the dose. The animals of both sexes that received the highest doses of extract had the lowest body weight on day 28.

#### Effect of oral administration of BTL on relative organ weight

**Fig. 9:**
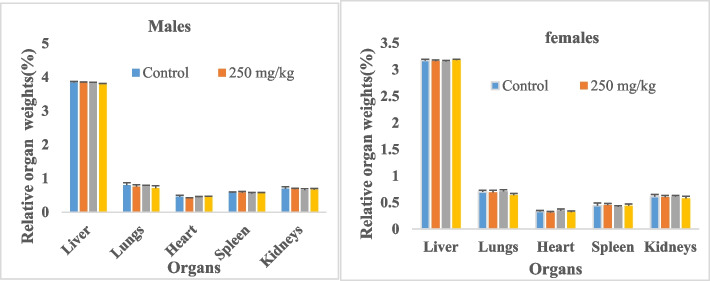
Effect of BTL on the relative organ weight of animals after 28 days of oral administration. Data are expressed as mean ± SD, *n* = 4. Values for a given group in a line followed by different symbols were significantly different (*p* < 0.05)

The effect of BTL on the relative organ weight of the animals after 28 days of administration is shown in Fig. 9. There were no significant changes in the liver, kidney, spleen, lungs, and heart weight in animals given different doses of extract compared to the control animals of both sexes.

#### Effect of oral administration of BTL on hematological parameters

Table 3 shows the effects of BTL on hematological parameters after 28 days of repeated oral administration. No significant (p ≥ 0.05) changes in hematological parameters were observed in animals of both sexes at all doses of the extract when compared to the animals in the control group.

#### Effect of oral administration of BTL on kidney function markers of animals

The effect of BTL on markers of rat kidney function after 28 days of administration is shown in Table 4. These results show that except for serum creatinine at 1000 mg/kg dose, which was significantly increased, no significant variation (*p* < 0.05) was observed with the other parameters measured compared to the animals in the control group.

**Table 4:** Effects of BTL on kidney function markers of animals

Sexes	Studies animals, doses, and values of investigated parameters				
	**Parameters**	**Doses of BTL and values of parameters**			
		**Control (0 mg/kg)**	**250 mg/kg**	**500 mg/kg**	**1000 mg/kg**
Females	S-Crea (mg/dL)	0.84 ± 0.02^a^	0.83 ± 0.02^a^	0.85 ± 0.01^a^	0.97 ± 0.03^b^
	S-Urea (mg/dL)	4.30 ± 0.42^a^	4.11 ± 0.37^a^	4.36 ± 0.33^a^	4.24 ± 0.40^a^
	U-Crea (mg/dL)	212.03 ± 19.72^ab^	180.49 ± 14.40^a^	226.77 ± 23.32^b^	224.01 ± 22.79^b^
	U-Proteins (g/dL)	0.04.03 ± 0.00^a^	0.03 ± 0.00^a^	0.04 ± 0.00^a^	0.04 ± 0.00^a^
	U-Urea (mg/dL)	482.13 ± 30.37^a^	465.41 ± 30.93^a^	493.03 ± 26.84^a^	461.55 ± 21.38^a^
Males	S-Crea (mg/dL)	0.83 ± 0.02^a^	0.80 ± 0.05^a^	0.80 ± 0.07^a^	0.79 ± 0.08^a^
	S-Urea (mg/dL)	3.62 ± 0.43^ab^	3.97 ± 0.13^b^	3.22 ± 0.45^a^	3.85 ± 0.35^ab^
	U-Crea (mg/dL)	257.10 ± 37.69^a^	246.09 ± 28.48^a^	255.03 ± 27.55^a^	248.12 ± 7.66^a^
	U-Proteins (g/dL)	0.04 ± 0.00^ab^	0.05 ± 0.00^ab^	0.04 ± 0.00^a^	0.06 ± 0.00^b^
	U-Urea (mg/dL)	451.08 ± 78.88^a^	501.25 ± 46.65^a^	439.74 ± 25.76^a^	496.46 ± 74.50^a^

Data are expressed as mean ± SD, *n* = 4. Values for a given group in a column followed by a different letter as a superscript are significantly different (*p* < 0.05). *S-Crea* serum creatinine, *U-Crea* urine creatinine, *S-Urea* serum urea, *U-Urea* urine urea, *U-Proteins* urine proteins

#### Effects of BTL on markers of the liver and cardiovascular function

The effect of sub-acute oral administration of BTL on biochemical markers of the liver and cardiovascular function is presented in Table 5. Analysis of the liver function markers revealed a significant (*p* < 0.05) decrease in alanine aminotransferase (ALT) and aspartate aminotransferase (AST) activities at 250 and 500 mg/kg in both sexes compared to those in the control group. Further analysis of cardiovascular markers revealed a significant (*p* < 0.05) increase in LDL cholesterol levels at 500 and 1000 mg/kg in females compared to the control group. However, in males, a significant (*p* < 0.05) increase in total and high-density lipoprotein HDL cholesterol levels at doses 500 and 1000 mg/Kg b. w. compared to those in the control group was observed. Similarly, sub-acute administration of methanol extract of BTL resulted in a significant decrease in LDL-cholesterol level at 250 mg/kg b. w. in males compared to the control group.

**Table 5:** Effect of BTL on the liver and cardiovascular markers of animals

Sexes	Studies animals, doses, and values of investigated parameters				
	**Parameters**	**Doses of BTL and values of parameters**			
		**Control (0 mg/kg)**	**250 mg/kg**	**500 mg/kg**	**1000 mg/kg**
Females	Proteins (g/dL)	5.12 ± 0.30^a^	4.87 ± 0.16^a^	4.79 ± 0.41^a^	4.97 ± 0.54^a^
	ALT (U/I)	40.46 ± 1.36^b^	33.46 ± 2.02^a^	36.36 ± 1.14^a^	46.37 ± 5.44^b^
	AST (U/I)	80.90 ± 1.43^a^	75.36 ± 2.60^a^	74.63 ± 1.60^a^	83.93 ± 5.80^b^
	TAG (mg/dL)	41.66 ± 2.9^ab^	47.96 ± 3.33^b^	35.55 ± 4.60^a^	45.92 ± 3.60^b^
	Cholesterol(mg/dL)	84.00 ± 7.38^a^	80.75 ± 3.27^a^	72.75 ± 6.76^a^	85.62 ± 6.35^a^
	LDL (mg/dL)	7.04 ± 0.87^a^	6.65 ± 0.39^a^	7.73 ± 0.22^b^	8.31 ± 0.20^c^
	HDL (mg/dL)	68.62 ± 6.61^ab^	64.50 ± 3.63^ab^	57.90 ± 7.02^a^	68.13 ± 2.52^b^
Males	Proteins (g/dL)	6.53 ± 0.51^a^	6.27 ± 0.36^a^	6.23 ± 0.44^a^	6.77 ± 0.34^a^
	ALT (U/I)	45.50 ± 1.06^b^	39.37 ± 0.57^a^	38.71 ± 1.95^a^	49.87 ± 5.10^b^
	AST (U/I)	130.37 ± 7.30^b^	78.53 ± 8.57^a^	80.28 ± 7.07^a^	134.74 ± 7.26^b^
	TG (mg/dL)	66.48 ± 5.32^b^	58.70 ± 8.79^ab^	50.00 ± 8.25^a^	71.85 ± 8.99^b^
	Cholesterol (mg/dL)	86.58 ± 1.70^a^	79.50 ± 5.91^a^	95.50 ± 4.73^b^	97.37 ± 2.49^b^
	LDL (mg/dL)	19.45 ± 0.81^b^	15.41 ± 0.42^a^	18.13 ± 1.16^b^	20.75 ± 0.91^b^
	HDL (mg/dL)	53.75 ± 1.84^a^	52.35 ± 3.59^a^	66.63 ± 5.51^b^	62.25 ± 2.39^b^

Data are expressed as mean ± SD, *n* = 4. Values for a given group in a Line followed by a different letter as a superscript were significantly different (*p* < 0.05). *ALT* alanine transaminase, *AST* aspartate transaminase, *HDL* high-density lipoprotein, *LDL* low-density lipoprotein, *TG* triglycerides

#### Histological sections of the liver and kidneys

**Fig. 10:**
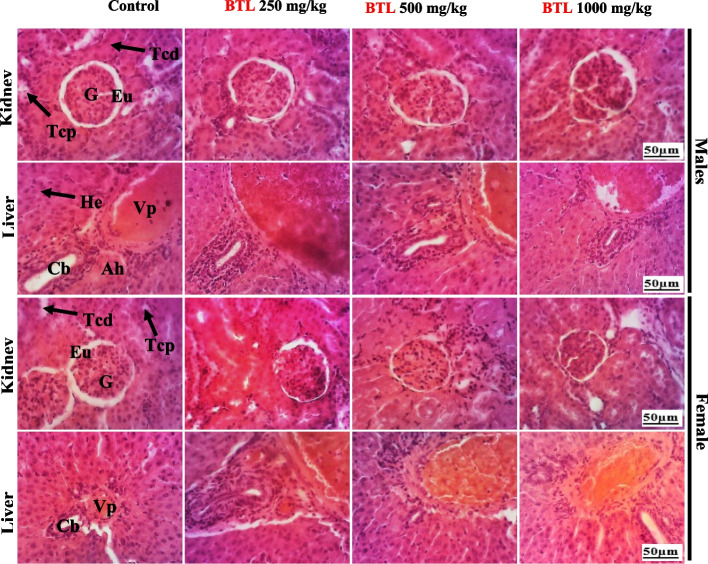
Histopathological analysis of the kidneys and the liver of males and females. Kidney: G = Glomerulus, Eu = Urinary space, Tcd = Distal bypass tubule, Tcp = Proximal bypass tubule. Liver: Vp = Portal vein, He = Hepatocyte, Cb = Bile canaliculus, Ah = Hepatic artery

Histopathological analysis of the liver and kidneys of both males and females revealed no abnormalities at all doses of BTL used in this test compared to the control animals (Fig. 10).

## Discussion

Cancer chemotherapy is seriously hampered by the rapid development of resistance to cytotoxic drugs. A good strategy for the new anticancer drug discovery should integrate the models of drug resistant cells [33, 39]. In the present study, the drug-resistant cancer cell lines included the P-gp-overexpressing CEM/ADR 5000 cells, the breast cancer resistance protein-expressing MDA-MB 231-*BCRP* cell lines, U87MG.*ΔEGFR* cells with mutation-activated *EGFR* gene (*ΔEGFR*) as well as the *p53* knockout HCT116*p53*^*−/−*^ cells [56–59]. To appreciate the importance of the studied samples as alternatives to combat cancer drug resistance, the drug sensitive counterparts of the above-mentioned resistant cells were used simultaneously. Cytotoxic medicines with the ability to tackle cancer drug resistance should show collateral or normal sensitivities of resistant cancer cells towards them [36, 37, 60, 61]. Moreover, efficient cytotoxic drugs should display IC_50_ values below 20 μg/mL for botanicals [62] or below 10 μM for phytochemicals [63]. Interestingly, the IC_50_ values below 20 µg/mL were recorded in the present work with BTL, BTLb, and BTLa on 1, 2, and 6 of the 9 tested cancer cell lines, respectively (Table 1). The IC_50_ values below 10 µM were obtained with compounds 2, 5, 6, 7, and 8 in at least 6/9 tested cancer cell lines (Table 2). This is a clear indication that these samples are potential cytotoxic agents. Besides, collateral or normal sensitivity of at least one model of the drug resistant cell line compared to their drug sensitive congener was reported with BTL, BTL a, and BTLb, compounds 2, 5, 6, 7, and 8 (Table 2). This also suggests that these samples could be useful in the fight against refractory cancers. To the best of our knowledge, the cytotoxicity of botanicals from *Bauhinia thonningii* as well as some of its phytoconstituents: 6-C-methylquercetin-3, 4'-dimethyl ether (1), 6-C-methylquercetin-3,7-dimethyl ether (2), 6-C-methylquercetin 3,7,3'-trimethyl ether (3), quercetin-3-*O*-_L_-rhamnopyranoside (5), quercetin-3-*O-β*-glucopyranoside (6), 6,8-C-dimethylkaempferol 3,7-dimethyl ether (7), 6,8-C-dimethylkaempferol-3-methyl ether (8) in such a broad panel of cancer cell lines including MDR phenotypes has been reported for the first time. However, the cytotoxicity of quercetin (4) was shown in CCRF-CEM cells and CEM/ADR5000 cells, with the IC_50_ values of 25 µM and 101 µM, respectively [64]. This corroborates the data obtained in the present study as compound 4 had a closer IC_50_ value of 27 µM in CCRF-CEM cells and a value above 50 µM in CEM7ADR5000 cells. Furthermore, the data obtained with *β*-sitosterol-3-*O-β*-_D_-glucopyranoside (10) corroborate the previous studies as this compound was found not active in the cancer cell lines tested in the current work with IC_50_ values above 100 µM [65]. In the present study, ursolic acid (9) showed a low cytotoxic effect in a single cancer cell line, CCRF-CEM with an IC_50_ value of 44.28 µM. This also corroborates other reports on this compound. For instance, Shyu reported an IC_50_ value of 75 µM for compound 9 in human hepatocellular carcinoma HuH7 cells [66]; the IC_50_ value of 7.1 μg/mL (or 15.57 µM) was also reported with compound 9 against MCF-7 cells [67]. Regarding the structure–activity relationship, the two isolated terpenoids (9 and 10) appear to have much lower cytotoxic effects than the flavonol derivatives 1 to 8 (Table 2). Within the flavonol derivatives, the types as well as the number of substituents in the flavonol moiety seem to influence the cytotoxic effects. Compound 4 with the lowest number of substituents (two hydroxyl groups) had the lowest spectrum of cytotoxic activity with IC_50_ values recorded in only two of the 9 tested cancer cell lines (Table 2). Compounds 1 and 3 are isomers with some differences found in the positions of *O*-methyl and hydroxyl groups in positions C7 and C4’. This resulted in a slight but not significant variation of the activity of the two compounds (Fig. 1; Table 2). The difference between compounds 5 and 6 is the presence of rhamnosyl substituent in position C3 (5) instead of glucosyl substituent (6). However, this change in the nature of the sugar did not seem to influence the cytotoxic effects of the two compounds as they had rather close inhibitory effects in all tested cancer cell lines (Fig. 1; Table 2). The difference between compounds 7 and 8 is the presence of *O*-methyl substituent in position C7 (7) instead of hydroxyl substituent (8) (Fig. 1). This substitution of *O*-methyl for hydroxyl seems to significantly increase the degree of the cytotoxicity of compound 8 (Table 2). IC_50_ values below 10 µM were obtained in 7/9 cancer cell lines with compound 8 and in only 5/9 for compound 7 (Table 2).

Apoptosis has been identified as one of the likely modes of action of several natural products including flavonoids [36, 61]. In this study, BTL and flavonoid 8 induced apoptosis in CCRF-CEM cells via caspase activation, MMP alteration, and increased ROS production. Though the mode of induction of apoptosis in CCRF-CEM cells by compound 8 (a kaempferol derivative) has not been reported yet, that of kaempferol is well-known in several cancer cell lines [68]. The results obtained in this work are therefore in conformity with the data on the mode of action of flavonols.

The toxicological profile of a substance is useful in the assessment of risks to humans [8, 69, 70]. However, adverse changes in animals have a strong predictive value for human toxicity especially as an active extract can be toxic to the host. It was important to assess the safety level of BTL in view of the promising cytotoxic data reported in this study. The acute toxicity evaluation of BTL revealed no deaths and no toxic signs in animals given an extract at a dose of 5000 mg/kg b. w. This indicates a high tolerance of rats to the extract. In accordance with this result, the LD_50_ of the BTL is above 5000 mg/kg b. w., which means that according to the Hodge and Sterner toxicity scale, this extract can be classified as being of low or no toxicity [71]. This result is in close agreement with the work of Olela et al. [72] who obtained an LD_50_ > 2000 mg/kg b. w. in the acute oral administration (in mice) of the aqueous and methanol extracts of *Bauhinia thonningii* bark harvested in Kenya. The sub-chronic administration of BTL at different doses for 28 days did not lead to any variation (*p* > 0.05) in food consumption. However, the decrease in weight growth in animals is not a sign of toxicity in view of the overall results observed as it is traditionally used in West Cameroon for its anti-obesity properties. This extract could therefore be beneficial for people suffering from obesity. According to Raji et al. [73], a plant extract with anti-obesity properties causes a decrease in the body weight of animals in toxicological studies.

Hematopoiesis is a vital process for life and can be affected by both conventional and herbal drugs. The assessment of hematological parameters in animal models provides information on the benefits or toxic manifestations of herbal extracts in the blood [74]. The results of the hematological evaluation in rats treated with BTL did not show significant changes in hematological parameters. This indicates that the different doses of the extract used do not affect hematopoiesis. The significant decrease (*p* < 0.05) in ALT and AST activity at doses of 250 and 500 mg/kg in animals of both sexes reflects the protective effect of the extract at these doses on the liver. Indeed, the botanicals with beneficial health effects act by reinforcing the selective permeability of the cell membranes. This led to a decrease in the level of enzymes and serum proteins [75]. The decrease in serum activities of its enzymes is a benefit to the liver and may reflect the protective effect of BTL on the liver. This non-hepatotoxic effect of BTL is confirmed by macroscopic and histopathological analysis of the liver that showed no lesions at all treatment doses in male and female animals and no significant variation in the relative liver weight. The kidney similarly to the liver is a key organ in toxicological assessment due to its marginal involvement in blood purification and excretion of waste products in the body. Its proper functioning can be assessed through certain parameters such as urinary proteins, urea, and creatinine. An increase in the urinary concentration of the latter will reveal the alteration of renal function [76]. The analysis of the results revealed no significant variation in urinary protein, urea and creatinine levels in rats of both sexes given different doses of BTL. Similarly, no pathological changes were observed in the histology of the kidneys of animals of both sexes. This indicates that BTL was not nephrotoxic at the doses used in this test. The administration of the plant extract in rats can also affect their lipid profile which is a set of parameters such as LDL-cholesterol, total cholesterol, and triglycerides that are mainly involved in the risk factors of cardiovascular diseases [77]. Based on the analysis of the lipid profile results, there was a significant decrease in LDL cholesterol levels at the dose of 250 mg/kg b. w. The LDL-cholesterol is the variant mostly occurring in pathological conditions, and its reduction as observed in this study reduces the risk of developing cardiovascular diseases.

## Conclusion

In the present work, the cytotoxicity of the methanol extract from the leaves of *Bauhinia thonningii* (BTL) as well as fractions (BTLa and BTLb) and their isolated phytoconstituents was evidenced on a panel of human cancer cell lines including MDR phenotypes. The isolated phytochemicals were 6-C-methylquercetin-3,4'-dimethyl ether (1), 6-C-methylquercetin-3,7-dimethyl ether (2), 6-C-methylquercetin 3,7,3'-trimethyl ether (3), quercetin (4), quercetin-3-*O*-_L_-rhamnopyranoside (5), quercetin-3-*O-β*-glucopyranoside (6), 6,8-C-dimethylkaempferol 3,7-dimethyl ether (7), 6,8-C-dimethylkaempferol-3-methyl ether (8), ursolic acid (9), and 3-*O*-*β*-_D_-glucopyranoside of *β*-sitosterol (10). The botanicals (BTL, BTL a, and BTLb) as well as compounds 2, 5, 6, 7, and 8 had promising cytotoxic effects. BTL and compound 8 induced apoptotic cell death in CCRF-CEM cells through caspase activation, alteration of MMP, and increased ROS production. BTL did not cause any adverse effects in rats after a single administration at 5000 mg/kg as well as the repeated dose of 250 mg/kg b. w. *Bauhinia thonningii* and its constituents are sources of cytotoxic drugs and deserve more in-depth studies to develop novel antiproliferative phytomedicine to fight cancer including resistant phenotypes.

### Supplementary Information

**Additional file 1: Figure S1-Figure S24.**

## Data Availability

All data generated or analyzed during this study are included in this published article and its supplementary information files.

## Abbreviations

1: 6-C-methylquercetin-3, 4'-dimethyl ether
2: 6-C-methylquercetin-3,7-dimethyl ether
3: 6-C-methylquercetin 3,7,3'-trimethyl ether
4: Quercetin
5: Quercetin-3-*O*-_L_-rhamnopyranoside
6: Quercetin-3-*O-β*-glucopyranoside
7: 6,8-C-dimethylkaempferol 3,7-dimethyl ether
8: 6,8-C-dimethylkaempferol-3-methyl ether
9: Ursolic acid
10: *β*-Sitosterol-3-*O-**β*-_D_-glucopyranoside
ALT: Alanine transaminase
AST: Aspartate transaminase
BCRP: Breast cancer resistance protein;
b. w.: Body weight
CC: Column chromatography
DMSO: Dimethyl sulfoxide
D. R.: Degree of resistance
EDTA: Ethylenediaminetetraacetic acid
EGFR: Epidermal growth factor receptor
EtOAc: Ethyl acetate
FITC: Fluorescein isothiocynate
H_2_DCFH-DA: 2’,7’-Dichlorodihydrofluorescein diacetate
HDL: High-density lipoprotein
HNC: Cameroon National Herbarium
IC_50_: Inhibitory concentration 50
JC-1: 5,5′,6,6′-Tetrachloro-1,1′,3,3′-tetraethylbenzimidazolylcarbocyanine iodide
m. p.: Melting point
*m/z*: Molecular weight
LDL: Low density lipoprotein
MDR: Multidrug-resistant
MMP: Mitochondrial membrane potential
P-gp: P-glycoprotein
PI: Propidium iodide
BTL: Methanol extract from the leaves of *Bauhinia thonningii*
BTL a and BTLb: Ethyl acetate and residual fractions from BTL
ROS: Reactive oxygen species
RRA: Resazurin reduction assay
SD: Standard deviation
S.I.: Selectivity index
sub-frs: Sub-fractions
TG: Triglycerides
WHO: World Health Organization
