# Supplementary material for: Cytotoxicity, acute and sub-chronic toxicities of the leaves of Bauhinia thonningii (Schumach.) Milne-Redh. (Caesalpiniaceae)
Source: BMC Complement Med Ther. 2023 Sep 27;23:341. doi: 10.1186/s12906-023-04172-9 (PMC10523748; doi:10.1186/s12906-023-04172-9)
Supplement: Supplementary file 1 — Additional file 1: Figure S1-Figure S24. [file 12906_2023_4172_MOESM1_ESM.docx]

Cytotoxicity, acute and sub-chronic toxicities of the leaves of *Bauhinia thonningii* (Schumach.) Milne-Redh. (Caesalpiniaceae)

Valaire Y. Matieta^a^, Armelle T Mbaveng^a,b^, Guy R. Sado Nouemsi^c^, Simplice B. Tankeo^a^, Gabriel T. Kamsu^a^, Paul Nayim^a^, Alain M. Lannang^c^_,_ İlhami Çelik^d^, Thomas Efferth^b*^, Victor Kuete^a,b**^

*^a^Department of Biochemistry, Faculty of Science, University of Dschang, Dschang, Cameroon*

*^b^Department of Pharmaceutical Biology, Institute of Pharmaceutical and Biomedical Sciences, University of Mainz, Staudinger Weg 5, 55128 Mainz, Germany*

*^c^ Department of Chemistry, Faculty of Science, University of Maroua, Maroua, Cameroon*

*^d^ Department of Chemistry, Faculty of Science, Eskişehir Technical University, Eskisehir, Turkey*

**Corresponding author:**

**Tel: (+49) 6131-3925751; Fax: (+49) 49-6131-3923752; E-mail:* [*efferth@uni-mainz.de*](mailto:efferth@uni-mainz.de)*; 55128 Mainz, Germany (Prof. Dr. Thomas Efferth)*

***Tel: +237 677355927; E-mail:* [*kuetevictor@yahoo.fr*](mailto:kuetevictor@yahoo.fr)*; P.O. Box 1499 Bafoussam, Cameroon (Prof. Dr. Victor Kuete)*

***Other author’s emails:***

*Matieta Yemene Valaire:* [*yvmatieta@yahoo.com*](mailto:yvmatieta@yahoo.com)

*Armelle T. Mbaveng: [armbatsa@yahoo.fr](mailto:armbatsa@yahoo.fr%20)*

*Simplice B. Tankeo: presidentankeo@yahoo.fr*

*Guy Raphael Sado Nouemsi:* [*raphael.n73@yahoo.com*](mailto:raphael.n73@yahoo.com)

*Gabriel Tchuente Kamsu: g.kamsu@yahoo.fr*

*Paul Nayim: nayimpaul@yahoo.fr*

*Alain Meli Lanang: [alainmeli@yahoo.com](mailto:alainmeli@yahoo.com)*

*İlhami Çelik:* [*ilcelik@gmail.com*](mailto:ilcelik@gmail.com)

**S1. General experimental procedure**

The proton and carbon (1D & 2D)-NMR (600/500 MHz) spectra were measured on Bruker AMX machine. The chemical shifts of proton and carbon were recorded based on the internal reference TMS (Tetramethylsilane) in δ (ppm). Moreover, coupling constants (J) were measured in Hz. High resolution mass spectra were obtained on QTOF Spectrometer equipped with a HESI source. The spectrometer was operated in positive mode (mass range: 100-1500, with a scan rate of 1.00 Hz) with automatic gain control to provide high-accuracy mass measurements within 0.40 ppm deviation using Na formate as calibrant. The following parameters were used for experiments: spray voltage of 4.5 kV, capillary temperature of 200 °C. Nitrogen was used as sheath gas (10 l/min). The spectrometer was attached to an Ultimate 3000 (Thermo Fisher, USA) UHPLC system consisting of LC-pump, Diode Array Detector (DAD) (λ: 190-600 nm), auto sampler (injection volume 5 μl) and column oven (40 °C). The separations were performed using a Synergi MAX-RP 100A (50 X 2 mm, 2.5µ particle size) with a H_2_O (+0.1 % HCOOH) (A)/acetonitrile (+0.1 % HCOOH) (B) gradient (flow rate 500 µL/min, injection volume 5 µL). Samples were analyzed using a gradient program as follows: 95 % A isocratic for 1.5 min, linear gradient to 100 % B over 6 min, after 100 % B isocratic for 2 min, the system returned to its initial condition (90 % A) within 1 min and was equilibrated for 1 min. UV/Visible spectroscopic data of compounds were recorded on an Evolution 300 spectrophotometer (Thermo Scientific). The purity of compounds and the monitoring of fractions were based on pre-coated silica gel TLC (Thin Layer Chromatography) plates supported on either plastic or aluminum sheets (E. Merck, F_254_). Spots were visualized on TLC with UV light (254 nm & 365 nm) on CN-6 UV spectrometer then sprayed with ceric sulphate and heated at about 90°C.

**S.2. Structural elucidation**

The phytochemical study of *Bauhinia thonningii* led to the isolation and identification of ten compounds whose structures are show in Figure 1. The spectral properties of these known compounds, including ^1^H NMR; ^13^C NMR and MHBC data, were identical to those previously described in the literature.

**Compound 1:** 6-C-methylquercetin-3,4'-dimethyl ether; yellow powder m.p. 195-197°C; *m/z* 345.09 for molecular formula C_18_H_16_O_7_; ^1^H NMR (600 MHz, CD_3_OD) *δ* 7.70 (d, *J* = 2.1 Hz, H-2'), 7.63 (dd, *J* = 8.4, 2.1 Hz, H-6'), 6.96 (d, *J* = 8.4 Hz, H-5'), 6.45 (s, H-8), 3.95 (*s*, 4'-OMe), 3.79 (*s*, 3-OMe), 2.09 (*s*, 6-CH_3_) and ^13^C NMR (150 MHz, CD_3_OD) *δ* 178.6 (C-4), 162.5 (C-7), 158.4 (C-5), 156.2 (C-2), 154.8 (C-9), 149.4 (C-4'), 147.5 (C-3'), 138.2 (C-3), 122.3 (C-6'), 121.6 (C-1'), 115.2 (C-5'), 111.5 (C-2'), 107.4 (C-6), 104.4 (C-10), 92.5 (C-8), 59.5 (3-OMe), 55.4 (3-OMe), 6.4 (6-Me) [1]

**Figure S1:** ^1^H NMR spectrum (600MHz, CD_3_OD) of compound (**1**)

**Figure S2:** ^13^C NMR spectrum (150MHz, CD_3_OD) of compound (**1**)

**Figure S3:** HMBC spectrum (600 MHz, CD_3_OD) of compound (**1**)

**Compound 2:** 6-C-methylquercetin-3,7-dimethyl ether; yellow powder; m.p. 195-197°C; *m/z* 344.32 for molecular formula C_18_H_16_O_7_; ^1^H NMR (600MHz, CD_3_OD) *δ* (ppm): 7.66 (d, *J* = 2.3 Hz, H-2'), 7.56 (dd, *J* = 8.4, 2.3 Hz, H-6') , 6.95 (d, *J* = 8.4 Hz, H-5'), 6.45 (s, H-8), 3.93 (s, 7-OCH_3_), 3.79 (s, 3-OCH_3_), 2.09 (s, 6-CH_3_). ^13^C NMR (600MHz, CD_3_OD) *δ* (ppm)**:** 156.7(C-2), 138.5(C-3), 178.7(C-4), 157.5(C-5), 107.1(C-6), 163.7(C-7), 89.1(C-8), 155.1(C-9), 104.5(C-10), 121.4(C-1'), 121.0(C-2'), 144.9(C-3'), 148.5(C-4'), 115.2(C-5'), 115.2(C-6'), 55.6(3-OCH_3_), 59.5(3'-OCH_3_), 7.0(6-CH_3_) [1, 2]

**Figure S4:** ^1^H NMR spectrum (600MHz, CD_3_OD) of compound (**2**)

**Figure S5:** ^13^C NMR spectrum (150MHz, CD_3_OD) of compound (**2**)

**Figure S6:** HMBC spectrum (600 MHz, CD_3_OD) of compound (**2**)

**Compound 3:** 6-C-methylquercetin-3,7,3'-trimethyl ether; yellow powder; m.p. 185-187°C; *m/z* 358.35 for molecular formula C_19_H_18_O_7_;^1^H NMR (600MHz, Acetone*-*d*_6_*) *δ* **(**ppm**):** 6.29 (d, *J* = 2.1 Hz, H-2'), 6.25 (dd, *J* = 8.4, 2.1 Hz, H-6') , 5.59 (d, *J* = 8.4 Hz, H-5'), 5.06 (s, H-8), 2.55 (s, 7- OCH_3_), 2.51 (s, 3-OCH_3_), 2.40 (s, 3'-OCH_3_), 0.69 (s, 6-CH_3_). ^13^C NMR (150MHz, Acetone*-*d*_6_*) *δ* (ppm): 156.9(C-1), 139.6(C-2), 179.4(C-3), 158.5(C-4), 109.4(C-5), 164.3(C-6), 90.8(C-7), 155.9(C-8), 106.3(C-9), 122.7(C-10), 112.1(C-1'), 147.8(C-2'), 149.7(C-3'), 115.2(C-5'), 123.5(C-6'), 60.8(7-OCH_3_), 56.7(3-OCH_3_), 56.6(3'-OCH_3_), 7.8(6-CH_3_) [1, 2].

**
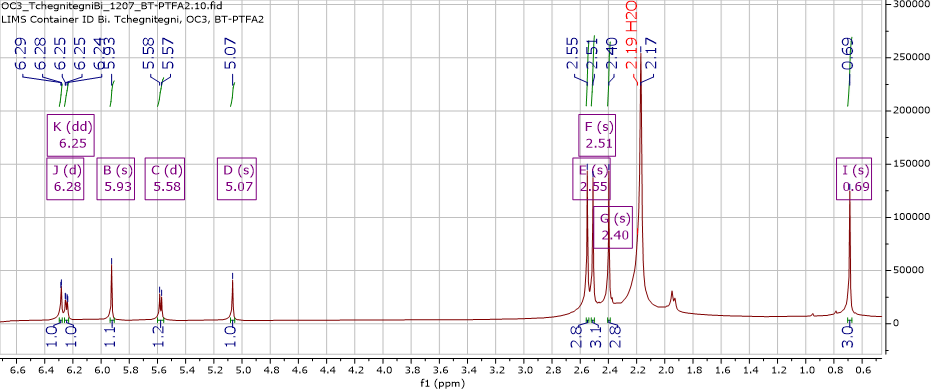
**

**Figure S7:** ^1^H NMR spectrum (600MHz, Acetone-d_6_) of compound (**3**)


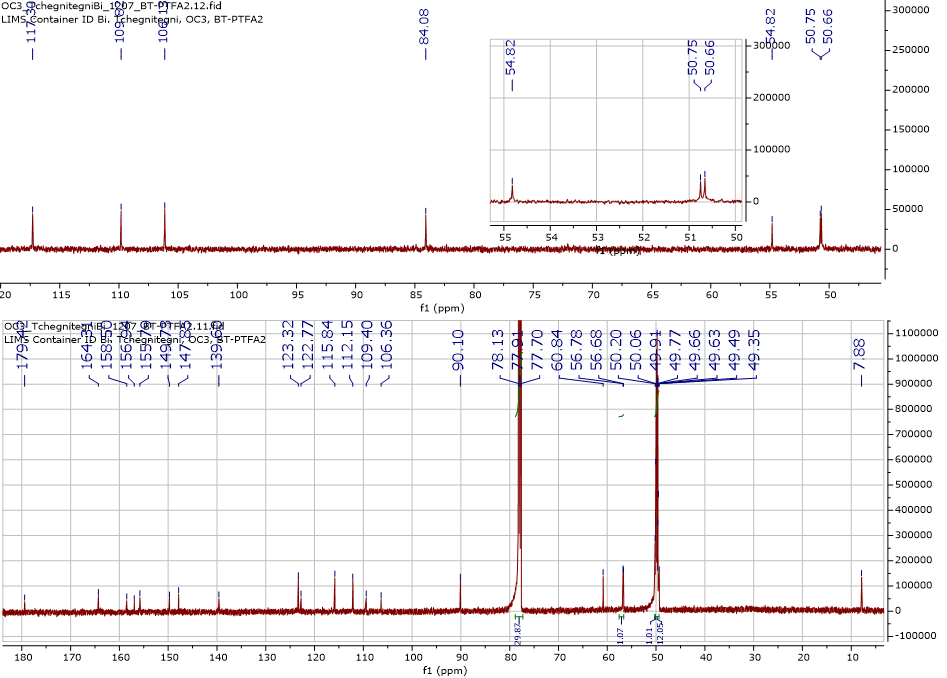
 **Figure S8:** ^13^C NMR spectrum (150MHz, Acetone-d_6_) of compound (**3**)

**Figure S9:** HMBC spectrum (600 MHz, Acetone-*d*_6_) of compound (**3**)

**Compound 4**: Quercetin; yellow powder; m.p. 314-317°C; *m/z* 302.24 for molecular formula C_15_H_10_O_7_;^1^H NMR (500 MHz, Acetone-*d*_6_) δ (ppm) 12.51 (5-OH), 10.81 (7-OH), 9.62 (4'-OH), 9.41 (3-OH), 9.34 (3'-OH), 7.68 (d, *J*=2.1 Hz, H-2'), 7.54 (dd, *J*=8.5, 2.1 Hz, H-6'), 6.89 (dd, *J*= 8.5, 2.1 Hz, H-5'), 6.41 (d, *J*= 2.0 Hz, H-8), 6.19 (d, *J*= 2.0 Hz, H-6); ^13^C NMR (125 MHz, Acetone-d_6_) δ (ppm): 147.2(C-2), 136.1(C-3), 176.2(C-4), 161.1(C-5), 98.6(C-6), 164.3(C-7), 93.7(C-8), 156.5(C-9), 103.4(C-10), 122.3(C-1'), 115.4(C-2'), 145.5(C-3'), 148.1(C-4'), 116.0(C-5'), 120.4(C-6') [2]

**Figure S10:** ^1^H NMR spectrum (500MHz, Acetone-d_6_) of compound (**4**)

**Figure S11:** ^13^C NMR spectrum (125MHz, Acetone-d_6_) of compound (**4**)

**Compound 5**: quercetin-3-*O*-_L_-rhamnopyranoside; yellow powder; m.p. 180-182°C; *m/z* 448.10 for molecular formula C_21_H_20_O_11_;^1^H NMR (500 MHz, CD_3_OD) δ (ppm) 7.35 (d, *J* = 2.1 Hz, H-2'), 7.32 ( dd, *J* = 8.3 and 2.2 Hz, H-6') , 6.93 (d , *J* = 8.3 Hz , H-5'), 6.27  (d , *J*=2.1 Hz , H-6), 6.45  (d, *J* = 2.6 Hz, H-8) ; 4.21 (dd, *J* = 3.4, 1.7 Hz, H-2''), 3,74 (dd, *J* = 9.3, 3.4 Hz, H-3''), 3.43 (dq, *J* = 9.4, 6.1 Hz, H-4''), 3.35 (d, *J* = 9.4 Hz, H-5''), 0.97 (d, *J* = 5.8 Hz, H-6''); ^13^C NMR (125 MHz, CD_3_OD) δ (ppm): 157.8 (C-2), 134.8 (C-3), 178.3 (C-4), 161.8(C-5), 98.6(C-6), 164.5(C-7), 93.6(C-8), 157.1(C-9), 104.4(C-10), 121.5(C-1'), 115.8(C-2'), 145.6(C-3'), 148.5(C-4'), 115.3(C-5'), 121.7(C-6'), 102.1(C-1''), 70.5(C-2''), 70.8(C-3''), 70.8(C-4''), 71.8(C-5’’) and 16.8(C-6’’) [1, 2]

**Figure S12:** ^1^H NMR spectrum (500MHz, CD_3_OD) of compound (**5**)

**Figure S13:** ^13^C NMR spectrum (125 MHz, CD_3_OD) of compound (**5**)

**Compound 6**: quercetin-3-*O*-*β*-glucopyranoside; yellow powder**;** m.p. 176-179°C; *m/z* 464.38 for molecular formula C_21_H_20_O_12_;^1^H RMN (500 MHz, CD_3_OD) *δ* (ppm) : 8.50 (d , *J* = 2.1 Hz , H-2'), 8.34 (dd, *J* = 8.3, 2.2 Hz, H-6'), 7.61 (d , *J* = 8.3 Hz ; H-5') ; 7.16 (d ; *J* = 2.6 Hz ; H-8) ; 6.96 (d ; *J*=2.1 Hz ;H-6) ; 6.03(d ; *J* = 7.6 Hz , H-1'') ;_’_4.26 (m, H-2''), 4.33 (q ; H-6a''), 4.21  (q, H-6b''), 4.46  (t, H-3'') ; 4.54  (m, H-4''), 4.19  (t , H-5''). ^13^C RMN (150 MHz, CD_3_OD) *δ* (ppm): 158.0(C-2), 134.9(C-3), 178.8(C-4), 162.3(C-5), 99.1(C-6), 165.3(C-7), 94.3(C-8), 157.9(C-9), 105.0(C-10), 123.3(C-1'), 117.1(C-2'), 145.4(C-3'), 149.3(C-4'), 115.8(C-5'), 122.5(C-6'), 103.9(C-1''), 74.4(C-2''), 72.4(C-3''), 69.3(C-4''), 76.8(C-5''), 61.3(C-6'') [1, 2]

**Figure S14:** ^1^H NMR spectrum (500MHz, CD_3_OD) of compound (**6**)

**Figure S15:** ^13^C NMR spectrum (125 MHz, CD_3_OD) of compound (**6**)

**Compound 7**: 6,8-C-dimethylkaempferol 3,7-dimethyléther**;** yellow powder m.p. 285-287°C; *m/z* 342.35 for molecular formula C_19_H_18_O_6_; ^1^H NMR (600 MHz, CD_3_OD) δ (ppm): 8.03 (d, *J* = 8.3 Hz, H-2'/H-6'), 6.95 (d, *J* = 8.8 Hz, H-3'/H-5'), 3.78 (s, 3-OCH_3_), 2.20 (s, 6-CH_3_) and 2.02(s, 8-CH_3_). ^13^C NMR (150 MHz, CD_3_OD) δ (ppm): 155.9(C-2), 137.9(C-3), 178.9(C-4), 157.0(C-5), 107.1(C-6), 160.5(C-7), 101.8(C-8), 152.2(C-9), 104.8(C-10), 121.7(C-1'), 130.0(C-2'), 115.2(C-3'), 160.7(C-4'), 115.2(C-5'), 130.0(C-6'), 59.4(3-OCH_3_), 7.3(6-CH_3_) and 6.7(8-CH_3_)[1, 2]

**Figure S16:** ^1^H NMR spectrum (600MHz, DMSO-d_6_) of compound (**7**)

**Figure S17:** ^13^C NMR spectrum (150MHz, DMSO*-d*_6_) of compound (**7**)

**Compound 8**: 6,8 -C- dimethylkaempferol-3-methyl ether; yellow powder; m.p. 250-253°C; *m/z* 228.09 for molecular formula C_18_H_16_O_6_; ^1^H NMR (600 MHz, DMSO-*d*_6_) δ (ppm): 12.78 (5-OH), 7.99 (d, *J* = 8.3 Hz, H-2'/H-6'), 6.97 (d, *J* = 8.5 Hz, H-3'/H-5'), 3.80 (s, 3-OCH_3_), 3.74 (s, 7-OCH_3_), 2.26 (s, 6-CH_3_) and 2.09 (s, 8-CH_3_); ^13^C NMR (150 MHz, DMSO-*d*_6_) δ (ppm): 156.5(C-2), 138.2(C-3), 179.0(C-4), 156.4(C-5), 113.0(C-6), 162.4(C-7), 109.0(C-8), 152.0(C-9), 107.5(C-10), 121.4(C-1'), 130.4(C-2'), 116.2(C-3'), 160.8(C-4'), 116.2(C-5'), 130.4(C-6'), 60.8(3-OCH_3_), 60.1(7-OCH_3_), 8.7(6-CH3) and 8.6(8-CH_3_) [1, 2]

**Figure S18:** ^1^H NMR spectrum (600MHz, CD_3_OD) of compound (8)

**Figure S19:** ^13^C NMR spectrum (150MHz, CD_3_OD) of compound (**8**)

**Compound 9**: Ursolic Acid; white powder; m.p. 283-285°C; *m/z* 456.71 for molecular formula C_30_H_48_O_3_; ^1^H NMR (500 MHz, C_5_D_5_N) δ (ppm) 5.40 (sl, H-12), 3.41 (dd, J = 11.0, 6 Hz, H-3), 2.60 (d, J = 12.0 Hz, H-18 ), 1.17 (s, H-23), 0.80  (s, H-24), 0.98  (s, H-25), 0.99(s, H-26), 1.19 (s, H-27), 0.96 (d, J = 6.0 Hz, H-29) and 0.88(d, J = 7, 1 Hz, H-30); ^13^C NMR (125 MHz, C_5_D_5_N) δ (ppm): 38.5(C-1), 27.4(C-2), 78.9(C-3), 39.1(C-4), 54.7(C-5), 17.9(C-6), 32.6(C-7), 38.9(C-8), 46.9(C-9), 38.2(C-10), 23.8(C-11), 125.5(C-12), 138.1(C-13), 41.6(C-14), 28.9(C-15), 23.8(C-16), 46.9(C-17), 52.3(C-18), 39.1(C-19), 39.8(C-20), 30.1(C-21), 36.5(C-22), 28.2(C-23), 16.8(C-24), 15.1(C-25), 16.1(C-26), 23.2(C-27), 180.7(C-28), 17.0(C-29) and 21.0(C-30) [1, 3]

**Figure 20:** ^1^H NMR spectrum (500MHz, Pyridine-d_5_) of compound **(9)**

**Figure 21:** ^13^C NMR spectrum (125MHz, Pyridine-d_5_) of compound **(9)**

**Compound 10**: 3-*O-ß*-D-glucopyranoside of *ß*-sitosterol; White powder; m.p. >212°C; *m/z* 456.71 for molecular formula C_35_H_60_O_6_; ^1^H NMR (500 MHz, C_5_D_5_N) δ (ppm) 5.31(sl, H-6), 4.21 (d, 8.0Hz, H-1’), 3.12 (m, H-12), 2.10 - 3.10 (m, H-2’- 6’), 0.98 (s, H-19), 0.89 (d, 6.5 Hz, H-21), 0.82 (d, 7.0 Hz, H-29), 0.81 (d, 7,0 Hz, H-26), 0.79 (d, 7,5 Hz, H-27), 0.63 (t, H-18); ^13^C NMR (125 MHz, C_5_D_5_N) δ (ppm): 36.6(C-1), 29.1(C-2), 77.2(C-3), 38.7(C-4), 140.8(C-5), 121.6(C-6), 31.8(C-7), 29.7(C-8), 50.0(C-9), 35.9(C-10), 20.1(C-11), 37.2(C-12), 42.3(C-13), 56.6(C-14), 23.0(C-15), 25.8(C-16), 55.8(C-17), 12.1(C-18), 19.5(C-19), 34.5(C-20), 19.3(C-21), 33.7(C-22), 24.3(C-23), 45.5(C-24), 28.2(C-25), 19.6(C-26), 19.0(C-27), 21.0(C-28), 12.1(C-29), 101.2(C-1'), 73.8(C-2'), 77.3(C-3'), 70.5(C-4'), 77.2(C-5') and 61.5(C-6') [1, 4].

**Figure 22:** ^1^H NMR spectrum (400 MHz, DMSO-d_5_) compound **(10)**

**Figure S23:** ^13^C NMR spectrum (100 MHz, DMSO-d_5_) of compound (**10**)

|  | R_1_ | R_2_ | R_3_ | R_4_ | R_5_ | R_6_ |
| --- | --- | --- | --- | --- | --- | --- |
| **1 :** | Me | Me | H | H | OH | OMe |
| **2 :** | Me | Me | Me | H | OH | OH |
| **3 :** | Me | Me | Me | H | OMe | OH |
| **4 :** | H | H | H | H | OH | OH |
| **5 :** | Rha | H | H | H | OH | OH |
| **6 :** | Glc | H | H | H | OH | OH |
| **7 :** | Me | Me | Me | Me | H | OH |
| **8 :** | Me | Me | H | Me | H | OH |

**Figure S24:** Chemical structure of compounds isolated from the leaves of B.thonningii

6-C-methylquercetin-3, 4'-dimethyl ether (**1**); 6-C-methylquercetin-3,7-dimethyl ether (**2**); 6-C-methylquercetin 3,7,3'-trimethyl ether (**3**); quercetin **(4)**; qercetine-3-*O*-L-rhamnopyranoside **(5)**; quercetine-3-*O*-L-rhamnopyranoside (**6**); 6,8-C-dimethylkaempferol 3,7-dimethyl ether **(7)**; 6,8-C-dimethylkaempferol-3-methyl ether **(8)**; ursolic acid **(9)** and 3-*O*-*β*-*D*-glucopyranoside of *β*-sistosterol **(10)**.

**References**

1. Nouemsi GRS, Jouda JB, Leutcha PB, Matieta VY, Tsague Tankeu VF, Ngnouzouba Kuete JR, Çelik İ, Kuete V, Sewald N, Lannang AM: A new flavonol derivative and other compounds from the leaves of *Bauhinia thonningii* Schum with activity against multidrug-resistant bacteria. *Nat Prod Res* 2022, 10.1080/14786419.2022.2128347:1-9.

2. Ibewuike JC, Ogundaini AO, Ogungbamila FO, Martin M-T, Gallard J-F, Bohlin L, Païs M: Piliostigmin, a 2-phenoxychromone, and C-methylflavonols from *Piliostigma thonningii*. *Phytochemistry* 1996, 43(3):687-690.

3. Mahato SB, Kundu AP: 13C NMR spectra of pentacyclic triterpenoids: A compilation and some salient features. *Phytochemistry* 1994, 37(6):1517-1575.

4. Waleguele CC, Mba'ning BM, Awantu AF, Bankeu JJK, Fongang YSF, Ngouela AS, Tsamo E, Sewald N, Lenta BN, Krause RWM: Antiparasitic constituents of *Beilschmiedia louisii* and beilschmiedia obscura and some semisynthetic derivatives (Lauraceae). *Molecules* 2020, 25(12):2862.
